# DNA sequencing, genomes and genetic markers of microbes on fruits and vegetables

**DOI:** 10.1111/1751-7915.13560

**Published:** 2020-03-24

**Authors:** Youming Shen, Jiyun Nie, Lixue Kuang, Jianyi Zhang, Haifei Li

**Affiliations:** ^1^ Institute of Pomology Chinese Academy of Agricultural Sciences/Laboratory of Quality & Safety Risk Assessment for Fruit (Xingcheng) Ministry of Agriculture and Rural Affairs/Quality Inspection and Test Center for Fruit and Nursery Stocks (Xingcheng) Ministry of Agriculture and Rural Affairs Xingcheng 125100 China; ^2^ College of Horticulture Qingdao Agricultural University Qingdao 266109 China; ^3^ Present address: No. 98, Xinghai South Street Xingcheng Liaoning Province 125100 China

## Abstract

The development of DNA sequencing technology has provided an effective method for studying foodborne and phytopathogenic microorganisms on fruits and vegetables (F & V). DNA sequencing has successfully proceeded through three generations, including the tens of operating platforms. These advances have significantly promoted microbial whole‐genome sequencing (WGS) and DNA polymorphism research. Based on genomic and regional polymorphisms, genetic markers have been widely obtained. These molecular markers are used as targets for PCR or chip analyses to detect microbes at the genetic level. Furthermore, metagenomic analyses conducted by sequencing the hypervariable regions of ribosomal DNA (rDNA) have revealed comprehensive microbial communities in various studies on F & V. This review highlights the basic principles of three generations of DNA sequencing, and summarizes the WGS studies of and available DNA markers for major bacterial foodborne pathogens and phytopathogenic fungi found on F & V. In addition, rDNA sequencing‐based bacterial and fungal metagenomics are summarized under three topics. These findings deepen the understanding of DNA sequencing and its application in studies of foodborne and phytopathogenic microbes and shed light on strategies for the monitoring of F & V microbes and quality control.

## Graphical abstract

The principles of three generations DNA sequencing were depicted. Whole genome sequencing and DNA markers of foodborne and plant fungal pathogens were summarized. Bacterial and fungal metagenomics studies on fruits and vegetables were concluded.

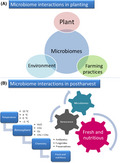

## Introduction

The requirements for the improvement of the quality and safety of horticultural fruits and vegetables (F & V) depend on a better understanding of microorganisms (Dean *et al.*, 2012; Olaimat and Holley, 2012; Siroli *et al.*, 2015). Foodborne pathogens pollute F & V under cultivation in diverse environments, the use of unsterilized agricultural inputs or improper storage. Such contamination can easily cause food poisoning (Olaimat and Holley, 2012). Phytopathogenic fungi cause plant diseases, postharvest deterioration and mycotoxin accumulation, which significantly affect yield, quality and market value (Dean *et al.*, 2012; Kumar *et al.*, 2017). However, several endophytes can also be used as biocontrol agents to provide beneficial conditions for cultivation and postharvest storage (Siroli *et al.*, 2015). Researchers are working to describe and control all of these microorganisms from farmland to consumers.

Microbial genome analysis relies strictly on DNA sequencing technology. The genome is the collection of DNA molecules, in which genes and variable sequences are arranged and provide the basic information for the formation of a microorganism. DNA sequencing, as a general technology applied in life science research, determines the nucleotide sequences of DNA strands. Over the past four decades, DNA sequencing technologies have rapidly developed and proceeded through three generations, resulting in the successful development of tens of platforms (Morey *et al.*, 2013). First‐generation sequencing (FGS) was developed in the mid‐1970s and was mainly based on Frederick Sanger's DNA chain‐termination sequencing method (Sanger *et al.*, 1974; Liu *et al.*, 2012). Next‐generation sequencing (NGS) was introduced in the 2000s, involving systems such as the Roche 454 pyrosequencing, Illumina Genome Solexa and Supported Oligo Ligation Detection (SOLiD) platforms (Liu *et al.*, 2012). Third‐generation sequencing (TGS) is the most recently introduced advance in this technology, which detects single and longer reads in real‐time with a high efficiency (van Dijk *et al.*, 2018). In parallel, DNA sequencing technologies have been applied in various genomic and phylogenetic studies (Rogers *et al.*, 2008; Morey *et al.*, 2013). First, DNA sequencing and whole‐genome sequencing (WGS) were applied in microbial studies. Sanger sequencing was first used to determine the genome of *phage X174* (5386 bp; Sanger *et al.*, 1977). Subsequently, Sanger et al. verified the sequencing procedure and determined the genome of *phage λ* (48 502 bp; Sanger *et al.*, 1983). In 1990, Goebel et al. reported the whole genome of *vaccinia* virus (192 kb), obtained by using the first automatic DNA sequencer, the AB370 system (Goebel *et al.*, 1990). In 1991, Bankier et al. reported the genome of a human cytomegalovirus (229 kb; Bankier *et al.*, 1991). In 1990, genomic research was initiated in *Escherichia coli* and *Saccharomyces cerevisiae* as model systems in preparation for the Human Genome Project. In 1995, Fleischmann et al. reported the genome of the first cellular microbe, *Haemophilus influenzae* Rd (1.83 Mb; Fleischmann *et al.*, 1995). After the AB370 DNA sequencer was upgraded to the AB3730xl system, microbial WGS was greatly promoted. Since then, the genomes of important microbes such as *E. coli* (Blattner *et al.*, 1997), *Salmonella enterica* (Parkhill *et al.*, 2001), *Listeria monocytogenes* (Glaser *et al.*, 2001), *Staphylococcus aureus* (Kuroda *et al.*, 2001), *Campylobacter jejuni* (Parkhill *et al.*, 2000) and *Shigella flexneri* (Jin *et al.*, 2002) have been widely reported. Currently, approximately 100 microbial genomes per day are being registered on the US National Center for Biotechnology Information (NCBI) platform.

Knowledge of DNA polymorphisms improves the understanding of microbial genetic specificity. The microbial genome shows various sequence differences or polymorphisms. Microbial DNA polymorphisms are the basis for explaining the specificity of phenotypes, evolution and taxonomy (Foley *et al.*, 2009). Methods such as the amplified fragment length polymorphism (AFLP), random fragment length polymorphism (RFLP), randomly amplified polymorphic DNA (RAPD), simple sequence repeat (SSR) and single nucleotide polymorphism (SNP) approaches are important for DNA polymorphism studies. Significantly, polymorphisms in bacterial ribosomal DNA (rDNA) *16S rRNA* genes and fungal internal transcribed spacers (ITSs) have been widely used in studies of microbial taxonomy and identification (Sun *et al.*, 2013). The 16S rDNA sequences of bacteria are relatively short, containing several conserved and hypervariable regions, and can provide taxonomic information for bacteria at the genetic level. Fungal rDNA contains tandem repeats of noncoding ITS regions. These ITS regions show a high level of polymorphism, and they are effective for fungal identification. Recently, improved sequencing technologies and powerful databases and software have promoted the development of sequence‐based identification methods. In metagenomics, 16S rDNA and ITS sequencing present significant benefits for characterizing overall bacterial and fungal communities.

Notably, DNA sequencing has promoted studies involving F & V microbial WGS, gene identification and specificity analysis. Genetic markers identified in studies of polymorphism have been widely used for polymerase chain reaction‐ (PCR) and chip‐based microbial detection (based, e.g. on conventional PCR, qPCR, multiplex PCR and gene chips; Lüth *et al.*, 2018). These detection methods can monitor foodborne pathogens and phytopathogens on F & V with good accuracy and acceptability (O'Connor and Glynn, 2010). In addition, metagenomic studies have revealed the bacterial and fungal communities on F & V by using 16S rDNA and ITS sequencing (Forbes *et al.*, 2017). The present review highlights the principles of DNA sequencing and outlines the WGS information and genetic markers of major bacterial foodborne pathogens and fungal phytopathogens on F & V. Common foodborne pathogenic species come from the *Escherichia*, *Salmonella*, *Staphylococcus*, *Listeria*, *Shigella* and *Campylobacter* genera (Table 1). Common phytopathogenic fungi come from the *Penicillium*, *Alternaria*, *Aspergillus*, *Fusarium*, *Botrytis*, *Colletotrichum*, *Monilinia* and *Trichothecium* genera (Table 2). Furthermore, NGS‐based metagenomic references have provided comprehensive data on key aspects of bacterial and fungal communities found on F & V (Table 3). These findings have deepened our understanding of DNA sequencing technology and its application in studies of foodborne and phytopathogenic pathogens, and they have shed light on methods for the microbial monitoring and quality control of F & V.

**Table 1:** Whole‐genome sequences of foodborne pathogens registered on NCBI platforms (up to 1 October 2019) and summary of the related genetic markers.

Species	ID number	Number of sequenced strains	Representative strain	Size (Mb)	GC%	Genes	Proteins	Specific genetic markers
*Escherichia*								
*E. coli*	ID167	17 952	K‐12 substr. MG1655 (Blattner *et al.*, 1997)	4.64	50.8	4498	4140	*fliC*, *Vt1*, *Vt2* (Gannon *et al.*, 1997); *uspA* (Osek, 2001); *lacZ* (Foulds *et al.*, 2002); *rfbE*, *eae*, *stx1*, *stx2* (Ooka *et al.*, 2009); ipaH (van den Beld and Reubsaet, 2012); *lacY*, *uidA* (Mendes Silva and Domingues, 2015); *PhoA* (Yang *et al.*, 2016); *cdtB* (Hassan *et al.*, 2018)
*E. albertii*	ID 1729	89	KF1	4.70	49.7	4823	4380	*cdtB* (Maheux *et al.*, 2014); *rpoB* (Lindsey *et al.*, 2015); *EAKF1_ch4033* (Lindsey *et al.*, 2017); *16S rDNA* (Grillova *et al.*, 2018)
*E. fergusonii*	ID 1877	18	ATCC 35469T	4.64	49.9	4618	4349	*yliE*, *EFER_1569*, *EFER_3126* (Simmons *et al.*, 2014); *EFER_0790* (Lindsey *et al.*, 2017)
*Salmonella*								
*S. enterica*	ID 152	11 215	CT18	5.13	51.9	4829	4473	*AceK*, *fliC*, *iagA*, *invA*, *oriC*, *sdf*, *sefA*, *ssaN*, *ssrA*, *STM2745*, *STM4492*, *16S rRNA* (Lee *et al.*, 2009; Postollec *et al.*, 2011); *sseL*, *spvC* (Peterson *et al.*, 2010); *hilA*, *fimA*, *hns* (Jeyasekaran *et al.*, 2011); *avrA*, *stn*, *stm* (Amin *et al.*, 2016); *spv*, *hut*, *fljB* (Alzwghaibi *et al.*, 2018)
*S. bongori*	ID1089	20	NCTC 12419	4.46	51.3	4382	4068	*fljB*, *gatD*, *invA* (Lee *et al.*, 2009); *fliC*, *gnd*, *mutS* (Soler‐Garcia *et al.*, 2014)
*Staphylococcus*								
*S. aureus*	ID 154	10 630	NCTC 8325	2.82	32.9	2872	2767	*mecA*, *nuc*, *femA‐SA*, *femA‐SE*, *orfx‐SCCmec*, *spa*, *gyrB*, *16S rRNA* (Hirvonen, 2014); *tuf*, *rpoB*, *gap*, *pyrH*, *ftsZ* (Song *et al.*, 2019); *sea*, *seb*, *sec*, *sed*, *see* (Omwenga *et al.*, 2019)
*S. epidermidis*	ID 155	670	ATCC 12228	2.56	32.1	2558	2482	*mecA*, *ermA*, *ermB*, *ermC*, *and msrA* (Martineau *et al.*, 2000); *hld*; *ica*, *agrA*, *sarA* (Frebourg *et al.*, 2000); *16S rDNA and tuf* (Kobayashi *et al.*, 2009); *femA‐SA*, *femA‐SE* (Jukes *et al.*, 2010); *atlE*, *gap and mvaA* (Kilic and Basustaoglu, 2011); *recN* (Iorio *et al.*, 2011); *adhesin fibrinogen binding protein* (Sunagar *et al.*, 2013); *hla/yidD and hlb* (Pinheiro *et al.*, 2015); *Gmk2*, *pta and SESB* (Osmani Bojd *et al.*, 2017); *icaA*, *aap*, *bhp* (Ribic *et al.*, 2017)
*S. lugdunensis*	ID2548	26	HKU09‐01	2.66	33.9	2567	2567	*Agr* (Dufour *et al.*, 2002); *rpoB* (Drancourt and Raoult, 2002); *16S rRNA* (Skow *et al.*, 2005); *Fbl* (Campos‐Pena *et al.*, 2014)
*S. saprophyticus*	ID1350	108	ATCC 15305	2.58	33.1	2523	2351	*16S rRNA* (Gaszewska‐Mastalarz *et al.*, 1997); *femA* (Vannuffel *et al.*, 1999); *hrcA* (Paiva‐Santos *et al.*, 2016)
*S. pseudintermedius*	ID3429	223	HKU10‐03	2.62	37.5	2517	2384	*SpsJ* (Verstappen *et al.*, 2017); *sps* (Phumthanakorn *et al.*, 2017)
*Listeria*								
*L. monocytogenes*	ID 159	3063	EGD‐e	2.94	38.0	3055	2867	*hly*, *iap*, *mpl*, *prfA*, *inlA*, *inlB*, *actA* (Gasanov *et al.*, 2005); *plcA*, *16S RNA* (Xu *et al.*, 2008)
*L. seeligeri*	ID 1246	6	SLCC3954	2.80	37.4	2790	2663	*23S rRNA*, *iap* (Paillard *et al.*, 2003); *lse24‐315* (Liu *et al.*, 2004); *16S rRNA* (Dalmasso *et al.*, 2010); *5'‐exonuclease* (Hage *et al.*, 2014)
*Shigella*								
*S. flexneri*	ID 182	480	301	4.83	50.67	4788	4313	*ipaH*, *plasmid DNA*, *ial*, *16S rRNA* (Warren *et al.*, 2006); she PAI (Farfan *et al.*, 2010); *invC*, *rfc* (Ojha *et al.*, 2013); *genome marker* (Sahl *et al.*, 2015; Kim *et al.*, 2017a)
*S. boydii*	ID 496	113	1221_SBOY	4.84	50.7	5125	4745	*ipaH*, *16S rRNA* (Warren *et al.*, 2006); *invC* (Ojha *et al.*, 2013); *wzy* (Radhika *et al.*, 2014); *genome marker* (Sahl *et al.*, 2015; Kim *et al.*, 2017a)
*S. sonnei*	ID 417	1338	4303	4.55	51.1	5275	4009	*ipaH*, *16S rRNA* (Warren *et al.*, 2006); *IS1* (Hsu *et al.*, 2007); *she PAI* (Farfan *et al.*, 2010); *invC*, *wbgZ* (Ojha *et al.*, 2013); *IpaBCD* (Farshad *et al.*, 2015); *genome marker* (Sahl *et al.*, 2015; Kim *et al.*, 2017a)
*S. dysenteriae*	ID 415	67	Sd197	4.56	50.9	4834	4294	*ial*, *virA*, *16S rRNA* (Warren *et al.*, 2006); *invC*, *rfpB* (Ojha *et al.*, 2013); *genome marker* (Sahl *et al.*, 2015; Kim *et al.*, 2017a)
*Campylobacter*								
*C. jejuni*	ID149	1615	NCTC 11168	1.64	30.5	1668	1572	*pDT1720* (Ng *et al.*, 1997); *CC2*, *CJ2* (Sails *et al.*, 2001); *ciaB*, *pldA*, *CDT* (Ghorbanalizadgan *et al.*, 2014); *GTPase gene*, *hip*, *16S rRNA*, *rrs*, *cdaF*, *porA*, *16S‐23S ITS*, *Hyp*, *cjaA*, *ceuE*, *hipO*, *mapA*, *ceuA*, *askD*, *glyA*, *lpxA*, *ccoN*, *ORF‐C sequence*, *rpoB*, *oxidoreductase gene*, *cdtA*, *pepT* (Frasao *et al.*, 2017); *flaA*, *cadF*, *racR*, *dnaJ*, *cdtB*, *and cdtC* (Oh *et al.*, 2017); *gyrA* (Sierra‐Arguello *et al.*, 2018); *ask*, *cdt* (Kabir *et al.*, 2019)
*C. coli*	ID1145	928	Aerotolerant OR12	2.03	30.8	2185	2058	*arsP*, *arsR*, *arsC*, *acr3*, *arsB* (Noormohamed and Fakhr, 2013); *GTPase gene*, *hip*, *ceuA*, *CCCH*, *cdtB*, *porA*, *16S‐23S ITS*, *ceuE*, *mapA*, *hipO*, *glyA*, *cadF*, *oxidoreductase gene*, *cdtA*, *pepT*, *50S gene*, *VS1* (Frasao *et al.*, 2017); *ceuE* (Rodgers *et al.*, 2017); *cadF*, *asp* (Pavlova *et al.*, 2016; Banowary *et al.*, 2018)

**Table 2:** Whole‐genome sequencing of phytopathogenic fungi registered on NCBI platforms (up to 1 October 2019) and summary of the related genetic markers.

Species	ID number	Number of sequenced strains	Representative strain	Size (Mb)	GC%	Genes	Proteins	Specific genetic markers
*Penicillium*								
*P. expansum*	ID11336	9	MD‐8	32.36	47.5	11 060	11 060	*Polygalacturonase* (Hesham *et al.*, 2011); *PatF* (Tannous *et al.*, 2015); *idh*, *18S*, *β‐tubulin*, *calmodulin* (De *et al.*, 2016; Rharmitt *et al.*, 2016); *ITS1‐ITS4* (Hammami *et al.*, 2017)
*P. digitatum*	ID13384	3	Pd1	26.05	48.9	8962	8961	*PdCYP51* (Hamamoto *et al.*, 2001); *β‐tubulin* (Oshikata *et al.*, 2013); *Pdcyt b* (Zhang *et al.*, 2009); *ITS* (Liu *et al.*, 2017); *RPB1*, *cmd* (Chen *et al.*, 2017b)
*P. griseofulvum*	ID43141	2	PG3	29.14	47.3	9630	9630	*ITS and IAO* (Shi *et al.*, 2011)
*P. citrinum*	ID40785	2	JCM 22607	34.00	45.9	–	–	*msdC* (Yoshida and Ichishima, 1995); *ITS* (Song *et al.*, 2018)
*P. italicum*	ID34360	3	GL‐Gan1	31.03	45.8	–	–	*ITS* (Youssef *et al.*, 2010); *RPB1‐1*, *cmd‐3* (Chen *et al.*, 2017b); *RPB1*, *RPB2* (Chen *et al.*, 2019)
*Alternaria*								
*A. alternata*	ID11201	6	SRC1lrK2f	32.99	51.4	13 577	13 466	*endopolygalacturonase* (Garganese *et al.*, 2016); *glyceraldehyde 3‐phosphate dehydrogenase (Gpd)*, *Alt a1* (Gherbawy *et al.*, 2018); *AaSdhB*, *AaSdhC*, *AaSdhD* (Lichtemberg *et al.*, 2018); *β‐tubulin* (Basim *et al.*, 2018); *ITS1*, *ITS4*, *histone 3* (Wang *et al.*, 2019)
*A. arborescens*	ID12091	5	EGS 39‐128	33.89	50.9	–	–	*ITS* (Lorenzini and Zapparoli, 2014); *endopolygalacturonase* (Garganese *et al.*, 2016); Alt a1, *calmodulin*, *plasma membrane ATPase* (Elfar *et al.*, 2018); *elongation factor*, *β‐tubulin*, *glyceraldehyde‐3‐phosphate dehydrogenase* (Somma *et al.*, 2019)
*A. brassicicola*	ID865	2	Abra43	31.04	50.8	–	–	*Cutinase A* (Gachon and Saindrenan, 2004); *Microsatellite locus ABS28* (Singh *et al.*, 2014); *ITS4*, *ITS5* (Gao *et al.*, 2014)
*A. solani*	ID65961	2	NL03003	32.78	51.3	–	–	*ITS1*, *ITS2* (Zur *et al.*, 2002); *Alt_a1*, *ITS‐ptAs* (Gu *et al.*, 2017); *histidine kinase (HK1)* (Khan *et al.*, 2018)
*A. tenuissima*	ID76065	7	FERA 1166	35.70	51.1	13 653	13 575	*ITS1*, *ITS4* (You *et al.*, 2014); *AM‐toxin* (Andersen *et al.*, 2006); *histone* (Kou *et al.*, 2014)
*Aspergillus*								
*A. flavus*	ID360	60	NRRL3357	36.89	48.3	13 485	13 485	*aflS*, *aflO* (Degola *et al.*, 2007); *ITS*, *pksA*, *omtA* (Yin *et al.*, 2009b); *β‐tubulin* (Zarrinfar *et al.*, 2015); *aflatoxin biosynthesis gene cluster* (Callicott and Cotty, 2015); *aflQ* (Mahmoud, 2015); *ITS1*, *ITS4* (Mylroie *et al.*, 2016); *aflR –aflJ intergenic region* (Atoui and El Khoury, 2017); *ITS*, *aflP(1)*, *aflM*, *aflA*, *aflD*, *aflP(3)*, *aflP(2)*, *aflR* (Al‐Shuhaib *et al.*, 2018); *norA*, *omtA* (Hua *et al.*, 2018)
*A. niger*	ID429	14	FDAARGOS_311	35.74	49.7	11 602	11 190	ITS (Gonzalez‐Salgado *et al.*, 2005); aclF1 (Tuntevski *et al.*, 2013); β‐tubulin gene (Mirhendi *et al.*, 2016); calmodulin gene (Das *et al.*, 2017); benA (von Hertwig *et al.*, 2018)
*A. parasiticus*	ID12976	3	SU‐1	39.47	48.1	8645	8645	*nor‐1*, *ver‐1*, *omt‐1*, *apa‐2* (Chen *et al.*, 2002); *aflR* (Somashekar *et al.*, 2004); *ITS* (Luo *et al.*, 2009); *aflC*, *norA* (Yin *et al.*, 2015); *aflR‐aflJ intergenic region* (Atoui and El Khoury, 2017)
*A. tubingensis*	ID18109	2	CBS 134.48	35.15	49.2	12 592	12 319	*ITS* (Medina *et al.*, 2005); *sequence‐characterized amplified region (SCAR)* (Giaj Merlera *et al.*, 2015); *calmodulin* (Palumbo and O'Keeffe, 2015); *β‐tubulin* (Mirhendi *et al.*, 2016)
*A. carbonarius*	ID947	1	ITEM 5010	36.15	51.6	11 735	11 478	*Calmodulin* (Palumbo and O'Keeffe, 2015); *ITS* (Tryfinopoulou *et al.*, 2015); *acOTApks*, *acOTAnrps*, *acpks*, *laeA*, *veA* (El Khoury *et al.*, 2016)
*A. westerdijkiae*	ID40399	1	CBS 112803 (Abdel‐Wahhab *et al.*, 2016)	36.07	50.2	–	–	*ITS* (Gil‐Serna *et al.*, 2009); *pks*, *p450‐B03* (Gil‐Serna *et al.*, 2011); *β‐tubulin* (Durand *et al.*, 2019)
*Fusarium*								
*F. oxysporum*	ID707	115	f. sp. lycopersici 4287 (Ma *et al.*, 2010)	61.39	48.4	27 347	27 347	*FOW1* (Li *et al.*, 2014); *IGS*, *TEF‐1α*, *β‐tubulin* gene (Kim *et al.*, 2017b); *Tfo1 insertion* (Ortiz *et al.*, 2017); *Bik* (Pugliese *et al.*, 2013); *ITS* (Wu *et al.*, 2019); *Ef1α* (Chen *et al.*, 2017a); *SIX4*, *SIX5* (Ayukawa *et al.*, 2016); *SIX1*, *SIX3* (Debbi *et al.*, 2018); *FEM1*, *HPEG* (van Dam *et al.*, 2018); *FocScF/FocScR* (Singh and Kapoor, 2018)
*F. solani (Nectria haematococca)*	ID 537	3	77‐13‐4 (Coleman *et al.*, 2009)	51.29	50.8	15 708	15 708	*FS1/FS2* (Casasnovas *et al.*, 2013); *EF‐1α* (Muraosa *et al.*, 2014); *ITS* (de Souza *et al.*, 2017); *RPB* (Chitrampalam *et al.*, 2018)
*F. fujikuroi*	ID 13188	15	IMI 58289 (Wiemann *et al.*, 2013)	43.83	47.5	14 943	14 810	*histone H3* (Steenkamp *et al.*, 1999); *MAT allele* (Steenkamp *et al.*, 2000); *ITS* (Llorens *et al.*, 2006); *gaoB* (Faria *et al.*, 2012); *β2‐tubulin* (Zhang *et al.*, 2015); *TEF 1‐α* (Carneiro *et al.*, 2017)
*F. verticillioides*	ID 488	4	7600 (Ma *et al.*, 2010)	41.84	48.7	20 574	20 574	*EF‐1α* (Wu *et al.*, 2016); *FUM1*, *FUM19* (Omori *et al.*, 2018); *ITS* (Jedidi *et al.*, 2018); *calmodulin* (Mulè *et al.*, 2004); *EGFP* (Gai *et al.*, 2018)
*F. proliferatum*	ID 2434	13	ET1 (Niehaus *et al.*, 2016)	45.21	48.1	16 509	16 143	*Calmodulin* (Mulè *et al.*, 2004); *ISR* (Borrego‐Benjumea *et al.*, 2014); *SSR* (Moncrief *et al.*, 2016); *tef‐1α*, *FUM1* (Galvez *et al.*, 2017); *FUM19* (Proctor and Vaughan, 2017); *ITS* (Jedidi *et al.*, 2018)
*F. graminearum*	ID 58	11	PH‐1 (Cuomo *et al.*, 2007)	36.67	48.3	13 334	13 334	*MGB* (Waalwijk *et al.*, 2004); *gaoA* (de Biazio *et al.*, 2008); *cyp51A*, *beta‐tubulin* (Yin *et al.*, 2009a); *TRI12* (Nielsen *et al.*, 2012); *MAT* (Demeke *et al.*, 2010); *PKS13* (Atoui *et al.*, 2012); *Tri7*, *Tri13* (Tralamazza *et al.*, 2016); *TRI* (Wang and Cheng, 2017); *ITS* (Jedidi *et al.*, 2018); *tef‐1α* (Garmendia *et al.*, 2018)
*Colletotrichum*								
*C. acutatum*	ID38530	2	1	52.13	51.7	–	–	*ITS* (Talhinhas *et al.*, 2002); *beta‐tubulin 2* (Talhinhas *et al.*, 2005); *CaInt2* (Jelev *et al.*, 2008); *BenA* (Polizzi *et al.*, 2011); *beta‐tubulin (TUB)*, *GAPDH* (Karimi *et al.*, 2019); *MAT1‐2* (Furuta *et al.*, 2017)
*C. gloeosporioides*	ID17739	6	TYU	53.01	49.6	–	–	*RAPD* (Pileggi *et al.*, 2009); *CgInt* (Lopes *et al.*, 2010); *β‐tubulin (TUB)*, *actin (ACT)*, *GPDH* (Ramdeen and Rampersad, 2013); *act*, *cal*, *gapdh*, *gs*, *ITS*, *tub2* (Sharma *et al.*, 2017)
*C. coccodes*	ID56076	2	NJ‐RT1	50.12	53.8	–	–	*RAPD* (Dauch *et al.*, 2003); *ITS* (Baysal‐Gurel *et al.*, 2014); *CAL*, *ITS*, *TUB2* (Silva *et al.*, 2018)
*C. fructicola*	ID57524	3	15060 (Aguilar‐Pontes *et al.*, 2018)	55.92	53.2	–	–	*ITS* (Li *et al.*, 2013); *ACT*, *CAL*, *CHS‐1*, *ITS*, *TUB*, *GAPDH* (Gan *et al.*, 2017)
*Monilinia*								
*M. fructicola*	ID54919	2	LMK 125	44.68	40.1	–	–	*MO368‐1*, *Laxa* (Cote *et al.*, 2004); *18S rDNA* (Fulton and Brown, 1997); *β‐tubulin* (Fan *et al.*, 2014); *ITS* (Guinet *et al.*, 2016); *laccase‐2* (Wang *et al.*, 2018b); *cytochrome b* (Ortega *et al.*, 2019)
*M. laxa*	ID56076	2	EBR‐Ba11b	41.84	40.2	–	–	*18S rDNA* (Fulton and Brown, 1997); *MO368‐1*, *Laxa* (Cote *et al.*, 2004); *ISSR and RAPD* (Fazekas *et al.*, 2014); *ITS* (Guinet *et al.*, 2016); *laccase‐2* (Wang *et al.*, 2018b); *SCAR* (Ortega *et al.*, 2019)
*M. fructigena*	ID66926	3	ASM290963v1	39.33	41.7	–	–	*18S rDNA* (Fulton and Brown, 1997); *MO368‐1*, *Laxa* (Cote *et al.*, 2004); *β‐tubulin* (Fan *et al.*, 2014); ITS (Guinet *et al.*, 2016); *laccase‐2* (Wang *et al.*, 2018b)
*M. polystroma*	ID66925	1	ASM290964v1	44.63	39.2	–	–	*MO368‐1*, *Laxa* (Cote *et al.*, 2004); *ITS* (Guinet *et al.*, 2016); *laccase‐2* (Wang *et al.*, 2018b)
*Botrytis*								
*B. cinerea*	ID494	4	B05.10	42.63	42.0	13 703	13 703	*EcoRI* (Rigotti *et al.*, 2002); *IGS*, *SCAR* (Suarez *et al.*, 2005); *IGS* (Diguta *et al.*, 2010); *G3PDH*, *HSP60*, *RPB2*, *NEP1*, *NEP2* (Fan *et al.*, 2015); *ITS*, *β‐tubulin* (Reich *et al.*, 2016); *necrosis*, *nep1* (Munoz *et al.*, 2016)

**Table 3:** Summary of 16S rDNA and ITS sequencing‐based microbiome community studies of fruits and vegetables.

Samples	Bacteria/fungi	Target organs	Study focus	Technology/sequencing platform	16S rRNA target/primers	Taxonomic resolution/focused taxonomy	References
Topics	Microbiomes differences between plant species/genotypes						
Apple	Bacteria	Flower	Apple flower Microbiome	Pyrosequencing	Primers 799 and 1115 reverse	Genera: *Acetobacter*; *Arthrobacter*; *Burkholderia*; *Buttiauxella*; *Deinococcus*; *Escherichia/Shigella*; *Hymenobacter*; *Knoellia*; *Lactobacillus*; *Methanosarcina*; *Pantoea*; *Terrimonas*; *Truepera*	Shade *et al.* (2013)
Apple, Grapes, Lettuce, Peach, Spinach and Tomato	Bacteria	Fruit; Leaf	Overall diversity and composition of bacterial communities, and their variations in fruits and vegetables	Pyrosequencing	Metabarcoding V4eV7 (universal primer 799F and 1115R)	Family: *Bacillaceae*; *Comamonadaceae*; *Enterobacteriaceae*; *Flavobacteriaceae*; *Leuconostocaceae*; *Microbacteriaceae*; *Micrococcaceae*; *Moraxellaceae*; *Nocadioidaceae*; *Oxalobacteraceae*; *Pseudomonadaceae*; *Rhizobiaceae*; *Sphingomonadaceae*; *Xanthomonadaceae*	Leff and Fierer (2013)
Arugula	Bacteria	Leaf	Arugula phyllosphere indigenous microbiome	Pyrosequencing	V3‐V8	Genera: *Caulobacterales*; *Rhizobaiales*; *Rhodobacterales*; *Sphingomonadales*; *Burkholderiales*; *Rhodocyclales*; *Enterobacteriales*; *Pseudomonadales*; *Xanthomonadales*	Cernava *et al.* (2019)
Basil	Bacteria	Leaf	Naturally bacterial community on basil leaves	Pyrosequencing	V1‐V3	Genera: *Arcicella*; *Chryseobacterium*; *Flavobacterium*; *Sphingobacterium*; *Altererythrobacter*; *Novosphingobium*; *Sphingobium*; *Sphingomonas*; *Herbaspirillum*; *Acinetobacter*; *Pseudomonas*; *Rheinheimera*; *Enterobacter*; *Erwinia*; *Klebsiella*; *Kluyvera*; *Pantoea*; *Rahnella*; *Raoultella*	Ceuppens *et al.* (2015)
Blueberry	Bacteria	Root	Soil properties and plant cultivars cooperatively shaped the variations in bacterial diversity and networks in the rhizosphere	Illumina sequencing	V4‐V5	Genera: *Azospirillum*; *Phenylobacterium*; *Rhodanobacter*; *Steroidobacter*; *Pseudomonas*; *Leteibacter*; *Acidothermus*; *Dongia*; *Rhizomicrobium*; *Pseudolabrys*; *Acidothermus*; *Rhodanobacter*; *Bradyrhizobium*; *Rhodoplanes*; *Pseudolabrys*	Jiang *et al.* (2017)
Cilantro, Cucumber, Mung, Bean and Sprout	Bacteria	Fruit, Leaf and Stem	Bacterial diversity differences	Illumina sequencing	V1‐V3	Genera: *Xanthomonas*; *Sphingobacterium*; *Stenotrophomonas*; *Klebsiella*; *Enterococcus*; *Corynebacterium*; *Braachybacterium*; *Cronobacter*; *Brevibacterium*; *Rhizobium*; *Weissella*; *Serratia*; *Acinetobacter*; *Pantoea*; *Pseudomonas*; *Enterobacteriaceae*; *Bacillus*; *Enterobacter*; *Staphylococcus*	Jarvis *et al.* (2018)
Grape	Bacteria	Leaf	Characterize the natural microbiome of grapevine	Pyrosequencing	V6	Genera: *Neisseriaceae*; *Sphingomonadaceae*; *Xanthomonadaceae*; *Veillonellaceae*; *Comamonadaceae*; *Leuconostocaceae*; *Moraxellaceae*; *Pseudomonadaceae*; *Enterobacteriaceae*; *Streptococcaceae*	Pinto *et al.* (2014)
Grape	Bacteria	Fruit	Geographical origin and cultivar of grape by microbiome composition in Northern Italy and Spain Vineyards	Illumina sequencing	V3‐V4	Genera: *Alphaproteobacteria*; *Gamaproteobacteria*; *Actinobacteria*; *Bacilli*; *Clostridia*; *Shingobactellia*; *Cytophagia*; *Blastocatellia*; *Phycisphaerae*; *Acidimicrobia*; *Thermoleophilia*; *Deltaproteobacteria*; *Solibacteres*; *Gemmatimonadetes*; *Actinobacteria*; *Cyanobacteria*	Mezzasalma *et al.* (2018)
Grape	Bacteria	Fruit	Bacterial communities of Grenache and Carignan grape varieties	Not given	V4	Genera: *Bacillus*; *Oenococcus*; *Acinetobacter*; *Erwinia*; *Streptococcus*; *Pseudomonas*; *Haemophilus*; *Enhydrobacter*; *Methylobacterium*; *Veillonella*; *Corynebacterium*; *Lactobacillus*; *Neisseria*; *Gluconobacter*; *Sphingomonas*; *Staphylococcus*; *Micrococcus*	Portillo *et al.* (2016)
Grape	Fungi	Fruit; Leaf	Microbial relationship between soil, leaves and fruits	Illumina sequencing	ITS1	Genera: *Hydropisphaera*; *Mycoarthris*; *Chaetomium*; *Fusarium*; *Acremonium*; *Phoma*; *Cryptococcus*; *Cladosporium*; *Guehomyces*; *Alternaria*	Zhang *et al.* (2017b)
Grape	Fungi	Leaf	Natural microbiome of grapevine	Pyrosequencing	ITS2 and D2	Genera: *Zoophthora*; *Pandora*; *Rhizopus*; *Mucor*; *Aureobasidiu*; *Sporormiella*; *Alternaria*; *Kurtzmanomyces*; *Colacogloea*, *Lewia*; *Ustilago*; *Puccinia*; *Cronartium*	Pinto *et al.* (2014)
Grape	Fungi	Fruit	Wine grape yeast from China	Not given	ITS1	Genera: *Zygosaccharomyces*; *Candida*; *Issatchenkia*; *Pichia*; *Sporidiobolus*; *Hanseniaspora*; *Cryptococcus*; *Pichia*; *Issatchenkia*; *Metschnikowia*	Li *et al.* (2010)
Grape	Bacteria	Leaf; Fruit	Microbial relationship between soil, leaves and grapes	Illumina sequencing	V5‐V7	Genera: *Kocuria*; *Microvirga*; *Flavobacterium*; *Sphingomonas*; *Nocardioides*; *Pseudomonas*; *Gaiella*; *Arthrobacter*; *Bacillus*; *Blastococcus*	Zhang *et al.* (2017b)
Grape	Bacteria	Fruit	Geography and cultivar differences affect wine grape microbiomes	Illumina sequencing	V4	Genera: *Acetobacter*; *Erwinia*; *Gluconobacter*; *Hymenobacter*; *Janthinobacterium*; *Klebsiella*; *Lactobacillus*; *Microbacteriaceae*; *Sporosarcina*; *Pseudomonadaceae*; *Sphingomonas*; *Leuconostocaceae*; *Moraxellaceae*; *Methylobacterium*	Bokulich *et al.* (2014)
Grape	Fungi	Fruit	Geography and cultivar differences affect wine grape microbiomes	Illumina sequencing	ITS1	Genera: *Cladosporium* spp.; *Botryotinia Fuckeliana*, *Penicillium*; *Davidiella*; *Aureobasidium*; *Hanseniaspora*; *Candida*	Bokulich *et al.* (2014)
Grape	Fungi	Fruit	Cultivar differences affect microbial communities	Illumina sequencing	ITS1	Genera: *Myrothecium*; *Epicoccum*; *Cryptococcus*; *Mortierella*; *Guehomyces*; *Fusanrium*; *Phoma*; *Cladosporium*; *Alternaria*	Zhang *et al.* (2017a)
Kiwifruit	Bacteria	Leaf and Flower	Plant species, organ and Psa infection in shaping bacterial phyllosphere communities	Illumina sequencing	V3‐V4	Genera: *Acinetobacter*; *Actinobacteria*; *Bacillus*; *Brevundimonae*; *Massillia*; *Methylobacterium*; *Pseudomonas*; *Sphingomonas*; *Streptococcus*	Purahong *et al.* (2018)
Spinach and Rocket	Bacteria	Leaf	Leaf mineral content related to microbial community structure	Illumina sequencing	V3	Genera: *Aeromonas*; *Bacillus*; *Citrobacter*; *Curtobacterium*; *Enterobacter*; *Lelliottia*; *Obesumbacterium*; *Pantoea*; *Providencia*; *Pseudomonas*; *Shigella*	Darlison *et al.* (2019)
Spinach and Rocket	Fungi	Leaf	Leaf mineral content related to microbial community structure	Illumina sequencing	V3	Genera: *Dothioraceae*; *Davidiellaceae*; *Tremellales*; *Incertae Sedis*; *Davidiellaceae*; *Cystofilobasidiaceae*; *Tremellales*	Chen *et al.* (2018)
Tomato	Bacteria	Flower, Fruit, Leaf, Root and Stem	Tomato microbial diversity reflect the ecology of pathogenicity	Pyrosequencing	V2	Genera: *Pseudomonas*; *Micrococcineae*; *Xanthomonas*; *MethyloBacterium*; *Rhizobium*; *Sphingomonas*	Ottesen *et al.* (2013)
Tomato	Fungi	Flower, Fruit, Leaf, Root and Stem	Tomato microbial diversity reflect the ecology of pathogenicity	Pyrosequencing	EF4 and Fung5	Genera: *Hypocrea*; *Aureobasidium*; *Cryptococcus*; *Chaetomium*; *Fusarium*; *Aspergillus*	Ottesen *et al.* (2013)

Topics	Regional environment and farming practices affect microbiomes						
Apple	Fungi	Fruit	Location‐related fungal differences reflect microbial quality	Illumina sequencing	ITS1	Genera: *Stibella*; *Paraphaeosphaeria*; *Phoma*; *Tilletiopsis*; *Cryptococcus*; *Aureobasidium*; *Acremonium*	Shen *et al.* (2018b)
Cabbage and Lettuce	Bacteria	Leaf	Microbial communities location differences	Pyrosequencing	V4‐V5	Genera: *Acinetobacter*; *Alistipes*; *Barnesiella*; *Rikenella*; *Anaerococcus*; *Aerosphaera*; *Vagococcus*; *Parabacteroides*; *Kluyvera*; *Peptoclostriduim*; *Lysinibacillus*; *Lactobacillus*; *Serratia*; *Macrococcus*; *Weisella*; *Exiguobacterium*; *Kosakonia*; *Candidatus Nardonella*; *Leclercia*; *Obesumbacterium*; *Streptococcus*; *Escherichia*; *Clostridium*; *Aeromonas*; *Staphylococcus*; *Lactococcus*; *Buttiauxella*; *Citrobacter*; *Pseudomonas*; *Enterococcus*; *Klebsiella*; *Bacillus*; *Enterobacter*; *Pantoea*	Higgins *et al.* (2018)
Grape	Fungi	Fruit	Fungal diversity in ferments associated with grape microbes	Pyrosequencing	NL1 and NL4	Genera: *Columnosphaeria*; *Davidiella*; *Cryptococcus*	Morrison‐Whittle *et al.* (2017)
Grape	Fungi	Fruit	Grape microbiome related to field origin and environmental conditions	Illumina sequencing	ITS1	Family: *Botryosphaeriaceae*; *Dothioraceae*; *Pleosporaceae*; *Trichocomaceae*; *Incertae Sedis*; *Saccharomycetaceae*; *Saccharomycodaceae*; *Hymenochaetales*; *Peniophoraceae*; *Chytridiomycota*	Mezzasalma *et al.* (2017)
Lettuce	Bacteria	Leaf	Bacterial communities on plant leaf surfaces related to time, space and environment	Pyrosequencing	V5‐V7	Genera: *Xanthomonas*; *Pantoea*; *Pseudomonas*; *Massilla*; *Bacillus*; *Erwinia*; *Alkanindiges*; *Acinetobacter*; *Naxibacter*; *Duganella*; *Flavobacterium*; *Exiguobacterium*; *Arthrobacter*	Rastogi *et al.* (2012)
Lettuce	Bacteria	Leaf	Biotic stress shifted structure and abundance of Enterobacteriaceae in the lettuce microbiome	Pyrosequencing	V4‐V5	Genera: *Trabulsiella*; *Serratia*; *Klubsiella*; *Yersinia*; *Rahnella*; *Pantoea*; *Escherichia*; *Enterobacter*; *Buttiauxella*; *Cedecea*; *Citrobacter*; *Erwinia*	Erlacher *et al.* (2015)
Maize	Bacteria	Leaf	Organic fertilization increased antibiotic resistome in phyllosphere	Illumina sequencing	V4‐V5	Genera: *Sphingomonas*; *Pseudomonas*; *Chryseobacterium*; *Curtobacterium*; *Methylobacterium*	Chen *et al.* (2018)
Mulberry	Fungi	Fruit	Soil fungal community borne related to genotypes and disease occurrence	Illumina sequencing	ITS1 and ITS2	Genera: *Humicola*; *Mortierella*; *Scleromitrula*; *Schizothecium*; *Sphaerulina*; *Lecidella*; *Cortinarius*; *Russula*; *Hymenochaete*; *Mrakia*	Yu *et al.* (2016)
Peach and Nectarines	Fungi	Fruit	Yeasts traceability of organic and conventional farming types	PCR‐DGGE/GATC Biotech	D1/D2	Species: *Pichia Kluyveri*; *Pichia Fermentans*; *Candida*; *Tremella Flava*	Bigot *et al.* (2015)
Tomato	Bacteria	Flower and Fruit	Insects introduce variability in bacterial diversity	Illumina sequencing	V1‐V3	Family: *Microbacteriaceae*; *Methylobacteriaceae*; *Rhizobiales*; *Alphaproteobacteria*; *Proteobacteria*; *Rhizobiaceae*; *Sphingomonadaceae*; *Comamonadaceae*; *Gammaproteobacteria*; *Shewanellaceae*; *Enterobacteriaceae*; *Pseudomonadales*; *Pseudomonadaceae*; *Xanthomonadaceae*	Allard *et al.* (2018)

Topics	Artificial treatment and quality control in storage and processing						
Apple	Fungi	Fruit	Long‐term cold storage changed fungal components	Illumina sequencing	ITS1	Genera: *Mucor*; *Phoma*; *Cryptococcus*; *Tilletiopsis*; *Aspergillus*; *Penicillium*; *Acremonium*; *Aureobasidium*	Shen *et al.* (2018a)
Apple	Fungi	Fruit	Mycotoxin contamination associated with fungal flora of apple core	AB3730xl sequencing	ITS1 and ITS4	Genera: *Alternaria*; *Penicillium*; *Aspergillus*; *Fusarium*; *Trichoderma*; *Epicoccum*; *Diplodia*; *Davidiella*; *Nigrospora*; *Paraconiothyrium*; *Bionectria*; *Botrytis*	Soliman *et al.* (2015)
Arugula, Broccoli, Cabbage, Cauliflower, Horseradish; Radish and Turnip	Bacteria	Leaf	Microbiomes of vegetables related to plant disease resistance and human health	Illumina sequencing	V4	Genera: *Sphingopyxis*; *Sphingomonas*; *Pseudoxanthomonas*; *Pedobacter*; *Flavobacterium*; *Pseudomonas*; *Acinetobacter*; *Sphingomonas*; *Leuconostoc*; *Acinetobacter*; *Janthinobacterium*; *Achromobacter*; *Methylobacterium*	Wassermann *et al.* (2017)
Asparagus	Bacteria	Stem	High‐hydrostatic pressure treatment for postharvest preservation	Pyrosequencing	V1–V3	Genera: *Streptococcus*; *Rubrobacter*; *Paucibacter*; *Janibacter*; *Escherichia*; *Clostridium*; *Citrobacter*; *Bacteroides*; *Propionibacterium*; *Brevibacterium*; *Sphingomonas*; *Pantoea*; *Bacillus*; *Tatumella*; *Rhodanobacter*; *Gammaproteobacteria*; *Halomonadaceae*; *Brenneria*; *Raoultella*; *Acetobacter*; *Pseudomonas*; *Proteobacteria*; *Serratia*; *Enterobacteriaceae*; *Rahnella*; *Acetobacteraceae*; *Leuconostoc*; *Gluconobacter*	del Arbol *et al.* (2016a)
Baby Spinach	Bacteria	Leaf	Bacterial communities changed by the chlorine dioxide (ClO2) treatment	Illumina sequencing	V3‐V4	Genera: *Bacillus*; *Pseudomonas*; *Massilia*; *Pantoea*	Truchado *et al.* (2018)
Bean and Radish	Bacteria	Seed	Bacteria community in the early plant developmental stages	Illumina sequencing	Not mentioned	Genera: *Pantoea*; *Pseudomonas*; *Enterobacer*; *Ervinia*; *Pantoea*; *Streptomyces*; *Arthrobacter*; *Bacillus*	Torres‐Cortes *et al.* (2018)
Cabbage	Bacteria	Leaf	Microbiota changes during cabbage ferment progress	Illumina sequencing	V3‐V4	Genera: *Leuconostoc*; *Lactobacillales*; *Bacillus*; *Chryseobacterium*; *Pseudomonas*; *Enterobacteriaceae*; *Enterobacteriaceae*; *Pseudomonas*; *Chryseobacterium*; *Pseudomonas*	Einson *et al.* (2018)
Carrot	Bacteria	Root	Postharvest gamma irradiation affects surface carrot bacterial community	Illumina sequencing	V4‐V5	Genera: *Pseudomonas*; *Yersinia*; *Janthinobacterium*; *Bacillus*; *Escherichia*; *Geobacillus*; *Bacillales*; *Pseudomonadaceae*; *Enterobacteriaceae*; *Ureibacillus*	Dharmarha *et al.* (2019b)
Cherry	Bacteria	Fruit	High‐hydrostatic pressure treatment affects bacterial community	Pyrosequencing	V1–V3	Genera: *Streptococcus*; *Rubrobacter*; *Paucibacter*; *Janibacter*; *Escherichia*; *Clostridium*; *Citrobacter*; *Bacteroides*; *Propionibacterium*; *Brevibacterium*; *Sphingomonas*; *Pantoea*; *Bacillus*; *Tatumella*; *Rhodanobacter*; *Gammaproteobacteria*; *Halomonadaceae*; *Brenneria*; *Raoultella*; *Acetobacter*; *Pseudomonas*; *Proteobacteria*; *Serratia*; *Enterobacteriaceae*; *Rahnella*; *Acetobacteraceae*; *Leuconostoc*; *Gluconobacter*	del Arbol *et al.* (2016b)
Cherry	Bacteria	Fruit	High‐hydrostatic pressure processing changed the microbiological quality and bacterial biodiversity	Pyrosequencing	V1‐V3	Genera: *Enterobacteriaceae*; *Rahnella*; *Acetobacteraceae*; *Leuconostoc*; *Gluconobacter*	Toledo del Arbol *et al.* (2016)
Clementines, Potatoes and Tomatoes	Bacteria	Fruit and Root	Food packaging materials affected microbial community	Illumina sequencing	V3‐V4	Phylum: *Proteobacteria*; *Firmicutes*; *Actinobacter*; *Bacteroidetes*; *Verrucomicrobia*; *Terenicutes*; *Plantomycetes*	Brandwein *et al.* (2016)
Grape	Bacteria	Fruit	Bacterial community and its temporal succession during the fermentation	Pyrosequencing	V1–V3	Genera: *Gluconobacter*; *Pedobacter*; *Spingomonas*; *Janthinobacterium*; *Pseudomonas*	Piao *et al.* (2015)
Grape	Bacteria	Fruit	Diversity and evolution of microorganisms during grape wine fermentation	Pyrosequencing	Not given	Genera: *Orbus*; *Bifidobacterium*; *Wolbachia*; *Acetobacter*; *Gluconacetobacter*; *Lactobacillales*; *Gluconobacter*	Portillo and Mas (2016)
Grape	Bacteria	Leaf and Stems	Human and animal pathogens related to foodborne diseases in the grapevine endosphere	Pyrosequencing	V5‐V9	Genera: *Propionibacterium*; *Staphylococcus*; *Clostridium*; *Burkholderia*	Yousaf *et al.* (2014)
Grape	Fungi	Fruit	Changes in microbiota and mycobiota during spontaneous fermentation	Pyrosequencing	ITS1‐5.8S–ITS2	Genera: *Nakazawaea*; *Hanseniaspora*; *Zygosaccharomyces*; *Starmerella*; *Aureobasidium*; *Botryotinia*; *Pichia*; *Penicillium*; *Cladisporium*; *Thelebolus*; *Geuhomyces*	Stefanini *et al.* (2016)
Grape	Fungi	Fruit	Microbial community in wine fermentation	Illumina sequencing	BITS and B58S3	Genera: *Alternaria*; *Aspergllus*; *Aureobasidium*; *Botryotinia*; *Brettanomyces*; *Candida*; *Cladosporium*; *Cryptococcus*; *Erysiphe*; *Hanseniaspora*; *Kluyveromyces*; *Lachancea*; *Metchnikowia*; *Meyerozyma*; *Mucor*; *Penicillium*; *Pichia*; *Rhodosporidium*; *Rhodotorula*; *Saccharomyces*; *Torulaspora*; *Wicherhamomyces*; *Zygosaccharomyces*	Sternes *et al.* (2017)
Grape	Fungi	Fruit	Composition and changes of the fungal population during fermentation	Pyrosequencing	ITS1	Genera: *Sporobolomyces*; *Periconia*; *Peniophora*; *Leptosphaerulina*; *Issatchenkia*; *Bathalimia*; *Diaporthe*; *Botrytis*; *Pithomyces*; *Phoma*; *Leptospherulina*; *Cryptococcus*; *Alternaria*; *Zygosaccharomyces*; *Metschnikowia*; *Diplodia*; *Cytospora*; *Candida*	Stefanini *et al.* (2017)
Grape	Fungi	Fruit	Wine fermentation affected by the metabolome	Pyrosequencing	ITS1	Genera: *Penicillium*; *Aspergillus*; *Botryosphaeria*; *Starmerella*; *Saccharomyces*; *Hanseniaspora*; *Cladosporium*; *Aureobasidium*	Wang *et al.* (2015)
Grape	Fungi	Fruit	Soil microbial composition shifts based on under‐vine management, but no corresponding changes in fruits	Illumina sequencing	ITS1	Genera: *Penicillium*; *Sporobolomyces*; *Coprinellus*; *Ischnoderma*; *Mycosphaerella*; *Occultifur*; *Pestalotiopsis*; *Tilletiopsis*	Chou *et al.* (2018)
Grape	Fungi	Fruit	Microbiome dynamics during spontaneous fermentations of sound grapes in comparison with sour rot and Botrytis infected grapes	PCR‐DGGE	515F/806R	Genera: *Aspergillus*; *Rhizopus*; *Saccharomyces cerevisiae*; *Starmerella*; *Penicillium*; *Zygosaccharomyces*; *Acetobacter*; *Ameyamaea*; *Gluconacetobacter*; *Gluconobacter*; *Tanticharoenia*; *Oenococcus*; *Botrytis*; *Cladosporium*; *Hanseniaspora*	Lleixa *et al.* (2018)
Grape	Fungi	Fruit	Yeast communities on the grape berries affect by fungicides	PCR‐DGGE	ITS1 and ITS4	Species: *Aureobasidium Pullulans*; *Candida* sp.; *Candida Zemplinina*; *Cryptococcus Carnescens*; *Cryptococcus Dimennae*; *Cryptococcus Flavescens*; *Cryptococcus Magnus*; *Cryptococcus* sp.; *Cryptococcus Victoriae*; *Cryptococcus Wieringae*; *Hansenispora Uvarum*; *Issatchenkia Terricola*; *Metschnikowia Pulcherrima*; *Pichia Fermentans*; *Pichia Membrannifaciens*; *Rhodosporidium Babjevae*; *Rhodotorula Glutinis*; *Rhodotorula Nothofagi*; *Saccharomyces cerevisiae*	Milanovic *et al.* (2013)
Grape	Fungi	Fruit	Diversity and evolution of microorganisms during grape wine fermentation	Pyrosequencing	Not given	Genera: *Hanseniaspora*; *Saccharomyces*; *Issatchenkia*; *Candida*; *Saccharomycodes*	Portillo and Mas (2016)
Lettuce	Bacteria	Leaf	Chitin amendment changed the microbial composition	Illumina sequencing	V3‐V4	Genera: *Cellvibrio*; *Sphingomonas*; *Pedobacter*; *Azospirillum*; *Taibaiella*; *Nitrospira*; *Streptomyces*; *Nocardioides*	Debode *et al.* (2016)
Lettuce	Bacteria	Leaf	Gamma irradiation changed the phyllosphere microbiota	Illumina sequencing	V4‐V5	Genera: *Pseudomonas*; *Ureibacillus*; *Escherichia*; *Bacillus*; *Symbiobacterium*; *Thermoactinomycetes*	Dharmarha *et al.* (2019a)
Lettuce	Bacteria	Leaf	Innovative washing solutions shifted the lettuce microbiota	Pyrosequencing	V1‐V3	Genera: *Flavobacterium*; *Pseudomonas*; *Pedobacter*; *Oxalabacteraceae*; *Comamonadaceae*; *Chryseobacterium*; *Agrobacterium*; *Sphingomonas*; *Janthinobacterium*; *Methylophilaveae*	Siroli *et al.* (2017)
Lettuce	Fungi	Leaf	Chitin amendment changed the microbial composition	Illumina sequencing	ITS2	Genera: *Morteriella*; *Lecanicillium*; *Pseudogymnoascus*; *Pesudeurotium*	Debode *et al.* (2016)
Maize	Fungi	Seed	Composition and variation in the fungal communities associated with seeds during storage	AB3730xl sequencing	ITS1/ITS4	Genera: *Aspergillus*; *Fusarium*; *Penicillium*; *Alternaria*; *Trichoderma*; *Chaetomium*; *Botryothichum*; *Cladosporium*; *Bipolaris*; *Rhizopus*	Xing *et al.* (2018)
Orange Peel	Fungi	Fruit	Dynamic of fungi communities during the storage	Illumina sequencing	ITS1/ITS4	Genera: *Cyphellophora*; *Epicoccum*; *Meyerozyma*; *Saccharomycopsis*; *Trichomerium*; *Colletotrichum*; *Meira*; *Cryptostroma*; *Alternaria*; *Zymoseptoria*; *Bryochiton*; *Talaromyces*; *Acaromyces*; *Cosmospora*; *Mycosphaerella*; *Pseudopithomyces*; *Paraphaeosphaeria*; *Strelitziana*; *Cyclocybe*; *Pichia*; *Aspergillus*; *Cladosporium*; *Hanseniaspora*; *Ceramothyrium*; *Schwanniomyces*; *Cystofilobasidium*; *Penicillium*; *Candida*; *Microidium*; *Wallemia*	Wang *et al.* (2018a)
Peanut	Fungi	Seed	Dynamic of fungi communities during the peanut storage	Illumina sequencing	ITS1	Genera: *Ascomycota*; *Talaromyces*; *Nectriaceae*; *Ceratobasidiaceae*; *Clonostachys*; *Enericella*; *Eurotium*; *Phizopus*; *Penicillium*; *Aspergillus*	Ding *et al.* (2015)
Pear	Bacteria	Fruit	Chlorine drenching and after controlled atmosphere storage affect bacterial populations	AB3730xl sequencing	F‐27, R‐1492	Genera: *Curtobacterium*, *Pseudomonas*, *Pantoea*; *Erwinia Billingiae*; *Frigoribacterium*	Duvenage *et al.* (2017)
Spinach	Bacteria	Leaf	Changes in spinach phylloepiphytic bacteria communities following minimal processing and refrigerated storage	Pyrosequencing	V4	Genera: *Deinococcus*; *Exiguobacterium*; *Sphingomonas*; *Methylobacterium*; *Rhizobium*; *Brevundimonas*; *Acinetobacter*; *Pseudomonas*; *Stenotrophomonas*; *Pantoea*; *Escherichia*; *Ralstonia*; *Naxibacter*; *Massilia*	Lopez‐Velasco *et al.* (2011)

## DNA sequencing

### First‐generation sequencing

**Fig. 1:**
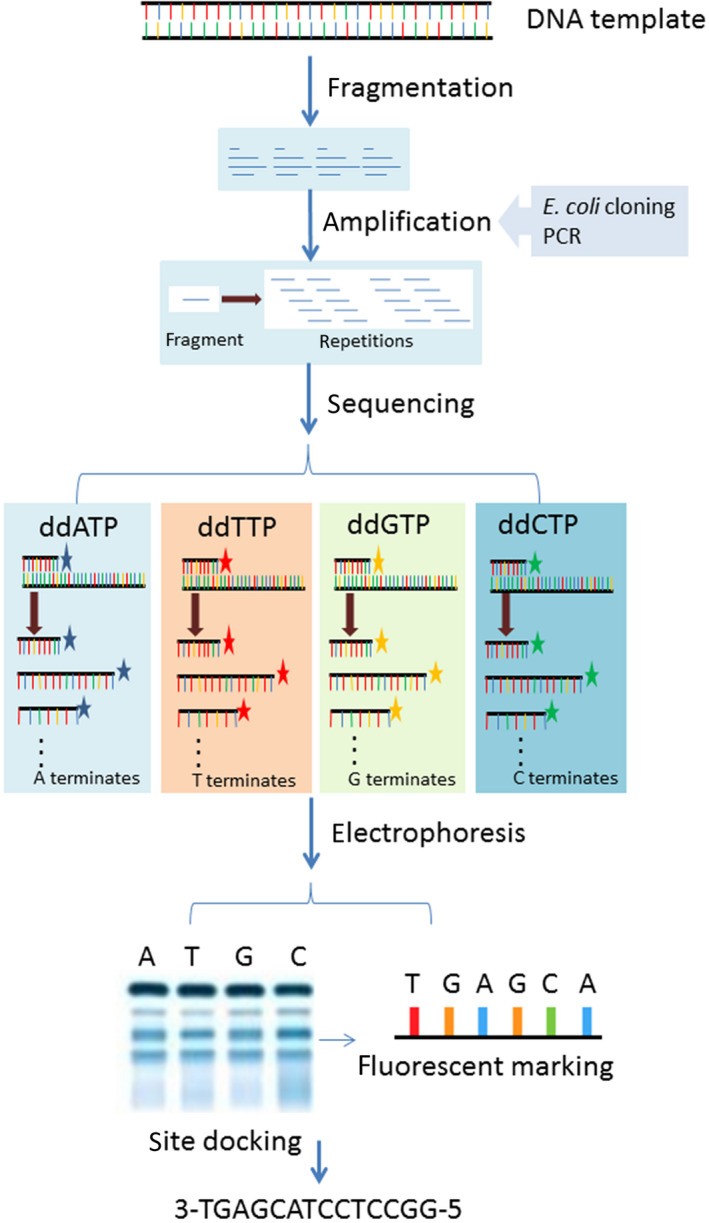
Schematic representation of first‐generation DNA sequencing.

The initial DNA sequencing methodology was developed in the mid‐1970s. Sanger et al. established a method for determining DNA sequences via primed synthesis with DNA polymerase (Sanger *et al.*, 1974; Sanger and Coulson, 1975). Progress in Sanger’s method was made after the introduction of chain‐terminating inhibitor dideoxyribonucleotides (ddNTPs; Sanger *et al.*, 1977). In the same year, Maxam and Gilbert reported a DNA sequencing approach based on chemical modification and specific cleavage (Maxam and Gilbert, 1977). The first‐generation DNA sequencing method of shotgun sequencing was developed based on the principles of both methods (Morey *et al.*, 2013). This technique involves the following steps (Fig. 1). A DNA template is broken down into fragments. Each DNA fragment is then amplified (initially by *E. coli* cloning and later by PCR). The amplicons of the DNA fragments are then sequenced in a system with four independent PCR arrays. In the four independent PCR assays, four chain‐terminating inhibitors (ddATP, ddTTP, ddGTP and ddCTP) are added. In the PCR assays, the majority of DNA polymerase reactions are conducted by adding dNTPs. However, in several reactions, ddNTPs are added for polymerization, thus terminating chain extension. As a result, DNA amplicons with different lengths are obtained. An electrophoresis method is used to separate the terminated DNA amplicons. The continuous DNA sequence is obtained by docking the ends of the electrophoresis‐separated products.

Automated DNA sequencing has been available since the introduction of fluorescently labelled ddNTPs and spectrum detection in the mid‐1980s. The first automated DNA sequencer (AB370) was announced by Applied Biosystems Co. in 1987 (Bankier *et al.*, 1991). The read length produced by the AB370 platform reached 600 bp, with detection of 96 bp per cycle and 500 kb per day. The AB370 system was upgraded to AB3730xl platform in 1995, with an improved read length (approximately 900 bases), speed (2.88 Mb per day) and accuracy (over 99.99%; Liu *et al.*, 2012). However, compared with NGS platforms, the AB3730xl system was far from meeting the needs for a higher speed, throughput and cost‐effectiveness.

### Next‐generation sequencing

Three NGS platforms were introduced during the first decade of the 21st century: the Roche 454 pyrosequencing, Illumina Genome Analyzer and SOLiD sequencing platforms. Compared with FGS, these NGS technologies perform multiparallel sequencing, which improves the throughput and speed and reduces costs.

**Fig. 2:**
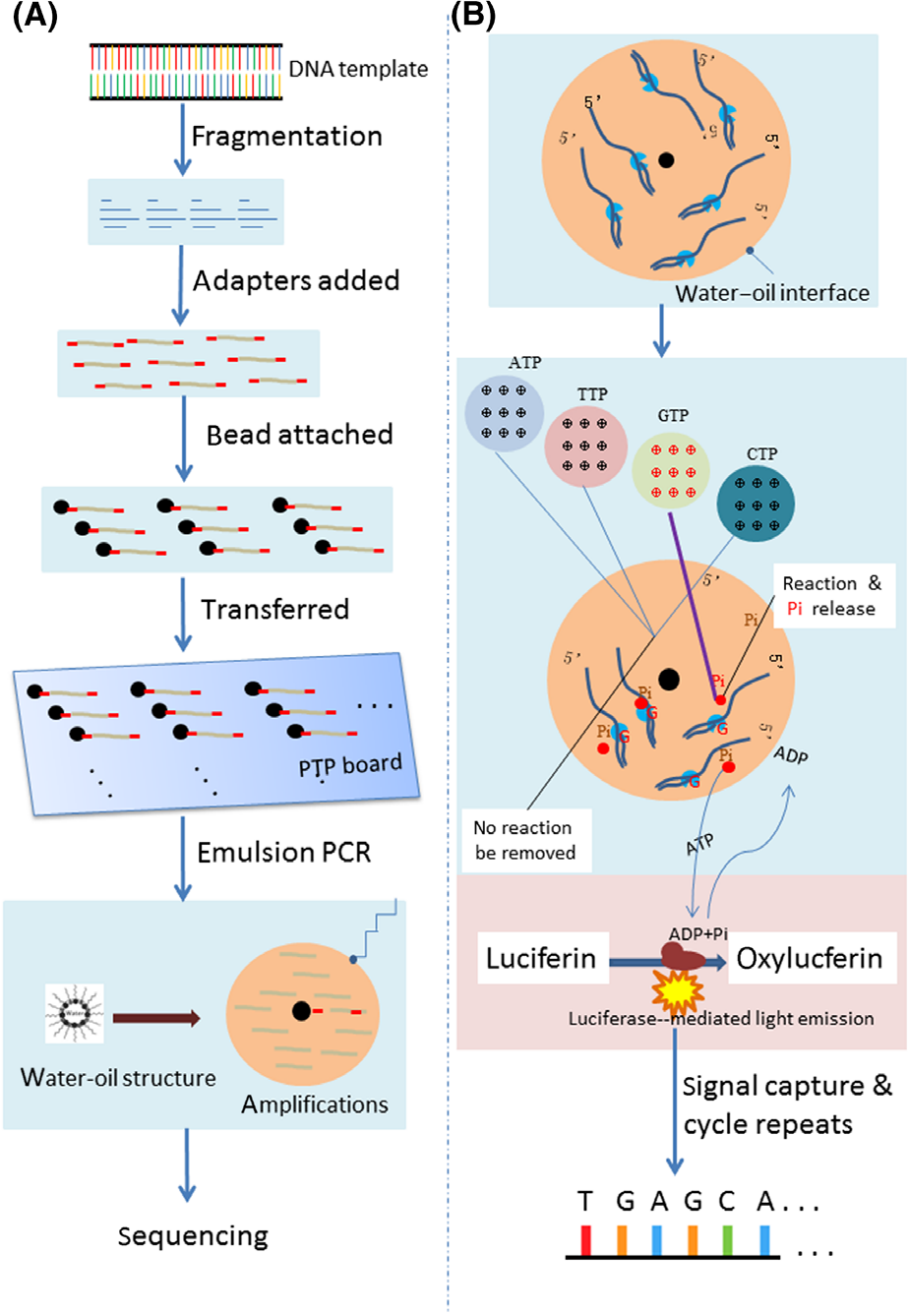
Schematic representation of Roche 454 pyrosequencing. Steps of DNA library preparation (A). Steps of pyrosequencing (B).

Roche 454 pyrosequencing was announced by the 454 Life Sciences Co. in 2005. Pyrosequencing is based on sequencing by synthesis and relies on the detection of the released pyrophosphate when a nucleotide polymerizes to the nascent DNA chain (van Dijk *et al.*, 2014). The template DNA is divided into 300‐ to 500‐base single‐stranded fragments (Fig. 2). Each DNA fragment was connected with two specific adapters at both ends and then attached to a 20 μm bead and transferred to a PTP hole (29 μm). The bead is emulsified to form a water‐in‐oil structure. The DNA fragment is amplified by performing emulsion PCR to form thousands of repetitions. In a single pyrosequencing cycle, four dNTPs (dATP, dGTP, dCTP and dTTP) are added to the PTP hole, and only one of them correctly matches the leading chain and is integrated into the nascent DNA. As soon as the correct dNTP is added and polymerized, the pyrophosphate is released to trigger luciferase‐mediated light emission. The signal is captured by a spectrum detector. The addition of the other three unpaired dNTPs does not generate a signal, and they will subsequently be removed. The combined data from hundreds of pyrosequencing cycles are used to generate DNA sequence reads. Thousands of PTP holes are arrayed on a PTP board. Therefore, numerous pyrosequencing reactions can be conducted simultaneously.

The Illumina Genome Analyzer was first introduced by Solexa in 2006 and purchased by Illumina in 2007. The Illumina platform is based on sequencing by synthesis (Morey *et al.*, 2013; Ghanbari *et al.*, 2015). The template DNA is first divided into 100‐ to 200‐base single‐stranded fragments. Both ends of the fragment are connected with an oligonucleotide adaptor. The two adaptors are complementarily matched with forward and reverse primers immobilized on a glass surface. The DNA fragment is amplified by performing bridge PCR to generate thousands of repeats. Repeats from a single DNA fragment form a separate strand by linearization. DNA sequencing is then performed by using four specific dNTPs. The dNTPs contain a specific cleavable fluorescent blocking group at the 3′‐OH end. In each sequencing cycle, the incorporation of a dNTP into the nascent DNA stimulates the release of a fluorescent signal, which is captured by a detector. Then, the blocking group on 3′‐OH is removed to continue the next sequencing step. Combined signals from hundreds of sequencing cycles are used to generate DNA sequence reads.

The SOLiD platform was introduced by Applied Biosystems in 2007. In contrast to sequencing by synthesis, SOLiD relies on the method of sequencing by ligation (Morey *et al.*, 2013). The NDA library preparation method is similar to Roche 454 pyrosequencing. The template DNA is first broken down into 30‐ to 50‐base single‐stranded fragments. Each DNA fragment linked with two adaptors at both ends and then attached to a 1 μm bead. The bead is immobilized on a glass slide. The DNA fragment is amplified by performing emulsion PCR. Sequencing is performed by using sixteen classes of 8‐base fluorescent nucleotide probes and 5 classes of primers. In the 8‐base probe, the 1st and 2nd positions at the 3′‐end can be occupied by any combination A, T, G or C, resulting in a total of 16 probes. The 8th probe position is linked with four fluorescent groups, which correspond to the first two nucleotides at the 3′‐end (mainly the 1st position). The 3rd and 4th positions of the probe can match any base. The 5th nucleotide is the cleavage site. Five universal primers match the continuous positions (n, n + 1, n + 2, n + 3 and n + 4) on the template DNA. Sequencing is initiated by hybridization. The first primer is hybridized to the template DNA (the initial site, n). Then, an 8‐base probe is introduced, which correctly matches the 1st and 2nd positions of the template DNA. Therefore, the fluorescent signal from the probe reflects the nucleotides of the 1st and 2nd positions (mainly the 1st position). After recording the signal, the fluorescent group is removed by cutting at the 5th position of the probe. Another probe matching the 6th and 7th positions of template is connected at the 5th cleavage site. Therefore, the second fluorescent signal reflects the 6th and 7th nucleotides. These steps are repeated until the ligation reaction is complete. The signals of the original consecutive fluorescent codes (n) are obtained, followed by melting. The second universal primer (n + 1) is used to obtain the corresponding fluorescent codes of the 2nd and 3rd positions, the 7th and 8th positions and so on. Then, three other universal primers (n + 2, n + 3 and n + 4) are used to obtain the three consecutive corresponding fluorescent codes. By combining the five signals (n, n + 1, n + 2, n + 3 and n + 4) of the colour codes and the read matrix, the original sequence of the template DNA can be calculated.

The three NGS platforms exhibit different characteristics (Liu *et al.*, 2012). Roche 454 sequencing produces a maximum read length of approximately 700 bases, which is longer than the read lengths generated by Illumina sequencing (150–200 bases) and SOLiD (30‐50 bases). In addition, Roche 454 sequencing is faster than Illumina or SOLiD sequencing. However, the Roche 454 platform presents an insufficient sequencing accuracy. Because dNTPs are not added to the last base of the DNA lagging chain, the last base of the sequence cannot be read. The Illumina sequencing platform exhibits the highest sequencing throughput and lowest operating cost per base. However, Illumina sequencing produces a shorter read length and therefore requires a greater sequencing depth. The two‐base coding and verification system of the SOLiD platform exhibits the greatest accuracy. However, the computing steps for the colour‐coding matrix and the combination of iterative data are complicated. Moreover, the shorter read length of this platform requires a greater sequencing depth. Several shortcomings commonly emerge in NGS platforms (Pushkarev *et al.*, 2009). Generally, the shorter read length of NGS requires that the template DNA be highly fragmented. This demands a greater sequencing depth and complex data computing for obtaining the overall reads. Second, PCR amplification is strictly required. However, PCR results are often inconsistent in regions such as those with a higher GC% or repeated hairpins. PCR amplicons also show variations in abundance under uniform PCR procedures (van Dijk *et al.*, 2018).

### Third‐generation sequencing

There are three leading TGS technologies: the HeliScope Single Molecule Sequencer (SMS), the single‐molecule real‐time (SMRT) approach and the Oxford Nanopore MinION sequencer (Reuter *et al.*, 2015; Lu *et al.*, 2016; van Dijk *et al.*, 2018). Compared with NGS, the fundamental improvement achieved by TGS is that a single strain or longer DNA molecules can be sequenced without PCR amplification. In addition, TGS technology shows real‐time performance, a higher throughput and reduced costs.

The HeliScope SMS was introduced by Helicos BioSciences in 2009 (Pushkarev *et al.*, 2009; Reuter *et al.*, 2015). The HeliScope SMS approach is based on sequencing by single‐molecule synthesis. The template DNA is first divided into single‐stranded fragments. Then, the 3′‐end fragment is linked to a poly‐A tail. Sequencing is performed on a HeliScope slide containing millions of flow cells. The flow cells contain a fixed with a poly T tail that both hybridizes with poly‐A sequence of the DNA template and provides a primer for DNA synthesis. Sequencing is initiated by the addition of the four fluorescently labelled and 3′‐OH‐blocked dNTPs, which is similar to Illumina sequencing. In each sequencing cycle, four dNTPs are added in turn. Once the correct dNTP is added and polymerized, the fluorescent signal is released. The signal is captured by a highly sensitive detection system. Then, the blocking group on the 3′‐OH is removed to begin the next sequencing step.

**Fig. 3:**
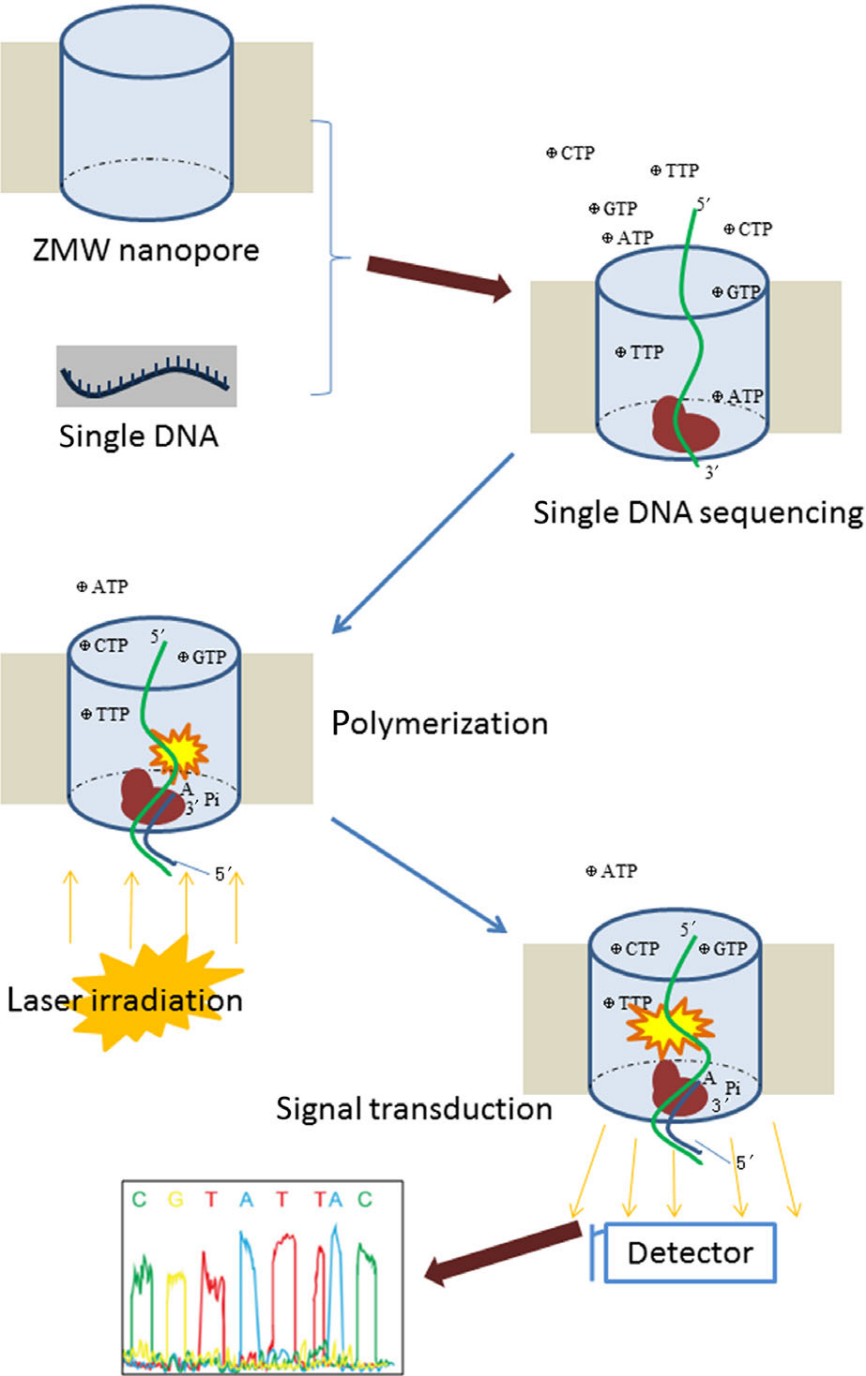
Schematic representation of SMRT sequencing.

The SMRT TGS platform was launched by Pacific Bioscience in 2011 (van Dijk *et al.*, 2018). SMRT sequencing is based on DNA synthesis, and the signal is captured via zero‐mode waveguide (ZMW) detection (Fig. 3). The ZMW nanopore is a channel with a diameter of 10 nm, which provides limited space for DNA polymerization. At the bottom of the ZMW nanopore, a DNA polymerase is immobilized. The template DNA is disintegrated into single‐stranded fragments of tens of kb. Both ends of a fragment are connected to two closed circular single‐stranded DNA adaptors. The DNA fragment is then introduced into the nanopore and ligated to the polymerase via either adaptor. Four fluorescently labelled and 3′‐OH‐blocked nucleotides are added to the reaction cells to start the synthesis process. Immediately after nucleotide polymerization, a fluorescent signal is generated. At the same time, laser irradiation of the nanopore is performed, and the fluorescent signal is amplified to a detectable level and transmitted to the nanopore‐external space. Thus, the undetectable fluorescent signal in the ZMW pore can be captured. Once the blocking group on 3′‐OH is removed, the next sequencing cycle continues. There are approximately 150 000 ZNWs in an SMRT unit, which is enough to obtain a sufficient throughput.

MinION sequencing was introduced by Oxford Nanopore Technologies in 2014 (Mikheyev and Tin, 2014). MinION sequencing is based on DNA electrophoresis, in which a‐haemolysin nanopores distributed across a semipermeable membrane serve as channels for DNA electrophoresis. Cyclodextrins covalently bind to the nanopores to increase nucleotide‐channel interactions. First, the template DNA is fragmented by using Covaris g‐TUBEs to form single‐stranded DNA fragments. The two ends of the DNA fragment are connected with two adapters. The lead adapter (Y adapter) is added to the 5′‐end, and the hairpin adapter (HP adapter) is added to the 3′‐end. An electric field is applied to both sides of the membrane to provide a driving force for DNA cross‐channel electrophoresis. Driven by voltage, DNA fragments enter and pass through the pores and interact with cyclodextrin in the process. Different nucleotide bases (A, T, G and C) interact with cyclodextrin differently and generate corresponding current waves. The ion current is measured and characterized to obtain the sequence of template DNA.

The three TGS platforms have different characteristics. The HeliScope SMS has not been widely used, because of its relatively slower speed, shorter read length and higher price. SMRT is the most commonly used TGS approach. In recent years, SMRT technology has been highly improved to achieve a sufficient throughput and cost‐effectiveness. The SMRT PacBio RSII platform produces a read length of 10–15 kb and a throughput of 0.5–1.0 Gb per run (van Dijk *et al.*, 2018). The read length of Nanopore MinION sequencing is similar to that of PacBio RSII. However, the error rate of Nanopore MinION (20–40%) is higher than that of PacBio RSII (10–15%; Lu *et al.*, 2016). However, the Nanopore MinION sequencer has attracted considerable interest due to its smaller size, cheaper equipment and lower running costs.

## WGS and genetic marker identification of related microbes on F & V

### WGS and genetic marker identification of related foodborne pathogen

**Fig. 4:**
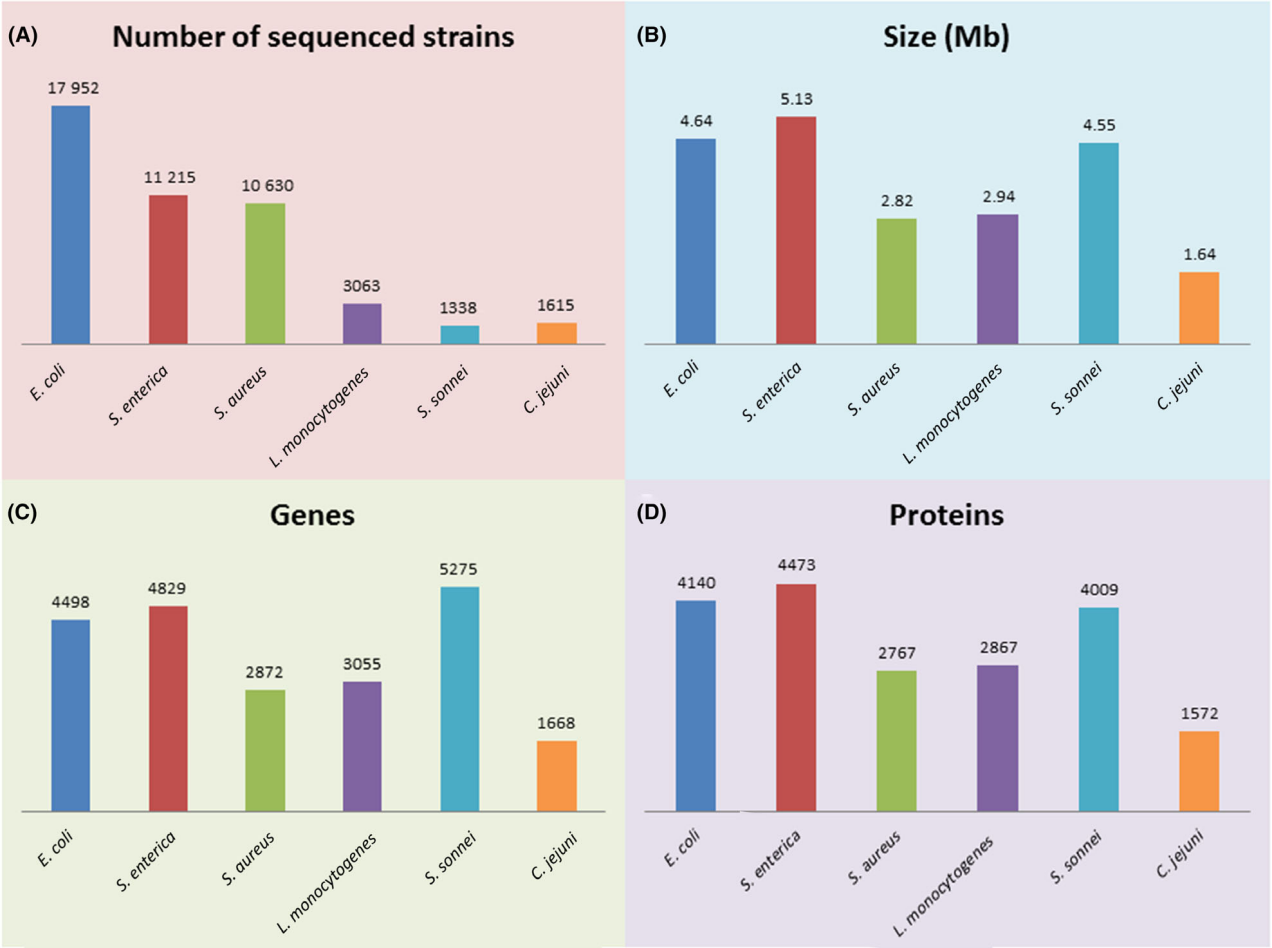
Genomic information for major foodborne pathogen species of *Escherichia coli* (*E. coli*), *Salmonella enterica* (*S. enterica*), *Staphylococcus aureus* (*S. aureus*), *Listeria monocytogenes* (*L. monocytogenes*), *Shigella sonnei* (*S. sonnei*) and *Campylobacter jejuni* (*C. jejuni*).

The *Escherichia* genus contains three species: *E. coli*, *E. albertii* and *E. fergusonii*. *E. coli* exhibits many strains, which usually colonized the intestines of humans and other mammals and are considered to be part of the intestinal flora. Gut *E. coli* can be released into the environment via feces. Therefore, the count of *E. coli* can reflect the extent of fecal contamination. In addition, several *E. coli* strains are serious opportunistic pathogens that cause food poisoning. To date, 17 952 *E. coli* genomes have been registered (Fig. 4), and approximately 1000 representative references have been summarized (NCBI genome ID 167). The first *E. coli* genome was obtained for strain K‐12 MG1655 by shotgun sequencing (Blattner *et al.*, 1997). Subsequently, Hayashi et al. reported the genome of the pathogenic strain *E. coli* O157:H7 RIMD 0509952 (Hayashi *et al.*, 2001). The genome of *E. coli* O157:H7 is 859 kb larger than that of strain K‐12 MG1655, and the comparison of their genomes showed extensive polymorphisms (Hayashi *et al.*, 2001). There are fewer reports of *E. albertii* and *E. fergusonii* as pathogens responsible for food poisoning. Genome sequencing has revealed 89 and 18 strains of *E. albertii* and *E. fergusonii* respectively (NCBI data). The representative *E. albertii* strain KF1 was the first to be sequenced and reported (NCBI genome ID 1729; Fiedoruk *et al.*, 2014). 16S rDNA sequencing has been used to distinguish *E. coli*, *E. albertii* and *E. fergusonii* (Maifreni *et al.*, 2013). Multiplex PCR based on the *cdgR*, *EAKF1_ch4033* and *EFER_0790* genes can efficiently distinguish *E. coli*, *E. albertii* and *E. fergusonii* (Lindsey *et al.*, 2017). For the identification of *E. coli*, the reported genetic markers mainly include *fliC*, *Vt1*, *Vt2* (Gannon *et al.*, 1997), *uspA* (Osek, 2001), *lacZ* (Foulds *et al.*, 2002), *rfbE*, *eae*, *stx1*, *stx2* (Ooka *et al.*, 2009), *ipaH* (van den Beld and Reubsaet, 2012), *lacY*, *uidA* (Mendes Silva and Domingues, 2015), *PhoA* (Yang *et al.*, 2016) and *cdtB* (Hassan *et al.*, 2018; Table 1). The *stx1*, *stx2* and *eae* genes have been reported as specific virulence markers for enterohemorrhagic *E. coli O157* (Franz *et al.*, 2007; Ooka *et al.*, 2009). The verotoxin genes (*VT1* and *VT2*) serve as markers for specific VT‐producing *E. coli* (Gannon *et al.*, 1997). *E. albertii* can be identified based on the specific 16S rDNA (Grillova *et al.*, 2018), cytolethal distending toxin (*cdtB*) gene (Maheux *et al.*, 2014) and cysteine biosynthesis gene (*EAKF1_ch4033*; Lindsey *et al.*, 2017) sequences. The regions of *yliE*, *EFER_1569* and *EFER_3126* are efficient for the multi‐PCR detection of *E. fergusonii* (Simmons *et al.*, 2014).

The *Salmonella* genus contains two pathogenic species, and *S. enterica* and *S. bongori*. *S. enterica* can be subdivided into six subspecies: *enterica* (I), *salamae* (II), *arizonae* (IIIa), *diarizonae* (IIIb), *houtenae* (IV) and *indica* (VI; Agbaje *et al.*, 2011; Lamas *et al.*, 2018). Alternatively, *S. enterica* can be subdivided into approximately 2,500 different serotypes according to somatic (O) and flagellar (H) antigens (Tindall *et al.*, 2005; Grimont and Weill, 2007). *S. enterica* subspecies I causes the majority of cases of foodborne diarrhoea (Sanchez‐Vargas *et al.*, 2011; Rezende *et al.*, 2016). *S. enterica* subspecies I exhibits approximately 1531 serotypes (Desai *et al.*, 2013; Lamas *et al.*, 2018). To date, approximately 11 215 strains of *S. enterica* have been registered (Fig. 4), and approximately 340 representative references concerning *S. enterica* genomic research have been summarized (NCBI genome ID 152). The genome of the representative *S. enterica* strain Typhi str. CT18 was the first to be reported (Parkhill *et al.*, 2001). Detailed genomic comparisons between *S. enterica typhi*, *E. coli* and *S. typhimurium* have been reported (McClelland *et al.*, 2001). The genomes of approximately 20 strains of *S. bongori* have been registered, among which the representative strain is *S. bongori* NCTC 12419 (NCBI genome ID 1089). The *sseL*, *spvC* (Peterson *et al.*, 2010), *avrA*, *stn*, *stm* (Amin *et al.*, 2016), *spv*, *hut*, *fljB* (Alzwghaibi *et al.*, 2018), *hilA*, *fimA* and *hns* (Jeyasekaran *et al.*, 2011) gene regions have been reported as markers for the *Salmonella* genus. In addition, multiplex PCR analysis based on the *fljB*, *gatD*, *invA*, *mdcA*, *stn* and *STM4057* genes is used for *Salmonella* genus identification (Lee *et al.*, 2009). For *S. enterica* species, the *AceK*, *fliC*, *invA*, *oriC*, *sdf*, *sefA*, *ssaN*, *STM2745*, *STM4492* and *16S rRNA* genes are widely used for PCR identification and detection (Lee *et al.*, 2009; Postollec *et al.*, 2011). The *fliC*, *gnd,* and *mutS* genes are used to distinguish *S. bongori* from other *Salmonella* pathogens (Soler‐Garcia *et al.*, 2014).

The *Staphylococcus* genus contains several species related to skin infection and food poisoning (mainly *S. aureus*, *S. epidermidis*, *S. lugdunensis*, *S. saprophyticus* and *S. pseudintermedius*)*.* Specifically, *S. aureus* is an important pathogen associated with toxin‐related food poisoning. As of recently, the genomes of approximately 10 630 *S. aureus* strains have been registered (Fig. 4), and approximately 300 representative references have been summarized (NCBI genome ID 154). The genomes of two *S. aureus* strains, N315 and Mu50, were the first to be determined by performing shotgun sequencing (Kuroda *et al.*, 2001). The characteristics of the genomes of potentially pathogenic species including *S. epidermidis*, *S. lugdunensis*, *S. saprophyticus* and *S. pseudintermedius* have been summarized (Table 1). Specific regions of *mecA*, *nuc*, *femA‐SA*, *femA‐SE*, *orfx‐SCCmec*, *spa*, *gyrB* and *16S rRNA* are used as markers for *S. aureus* identification (Hirvonen, 2014). Based on polymorphisms in the *femA* gene, *S. aureus* and *S. epidermidis* can be differentiated (Jukes *et al.*, 2010). Recently, multilocus sequence typing (MLST) was performed on *femA*, *tuf*, *rpoB*, *gap*, *pyrH* and *ftsZ* to identify *Staphylococcus* strains accurately (Song *et al.*, 2019). The staphylococcal enterotoxin (*se*) genes of *sea*, *seb*, *sec* and *see* have been used to monitor toxic *S. aureus* in food (Omwenga *et al.*, 2019).

The *Listeria* genus contains the pathogenic species *L. monocytogenes* and *L. seeligeri*. *L. monocytogenes* is an important foodborne pathogen that contaminates various F & V and causes human *listeriosis* (Buchanan *et al.*, 2017). About the genomes of 3063 strains of *L. monocytogenes* have been registered to date (Fig. 4), and nearly 80 genomic references have been summarized (NCBI genome ID 159). The genome of the representative *L. monocytogenes* strain EGD‐e was the first to be obtained (Glaser *et al.*, 2001). *L. seeligeri* is reported less often in food, and its genomic information is summarized in Table 1. Methods for *L. monocytogenes* identification have been reviewed previously (Gasanov *et al.*, 2005; Välimaa *et al.*, 2015). PCR‐based detection has mainly been performed for the *hly*, *iap*, *mpl*, *prfA*, *inlA*, *inlB*, *actA* (Gasanov *et al.*, 2005), *plcA* and *16S RNA* genes (Xu *et al.*, 2008).

The *Shigella* genus contains four foodborne pathogens, *S. flexneri*, *S. boydii*, *S. sonnei* and *S. dysenteriae* (Warren *et al.*, 2006). *Shigella* pathogens contaminate a variety of foods and exhibit diverse occurrences and different epidemiologies (Warren *et al.*, 2006; Levin, 2009; Lin *et al.*, 2010). *S. dysenteriae* serotype 1 causes deadly epidemics, *S. flexneri* causes endemic infection, foodborne diseases associated with *S. boydii* occur mainly in developing countries, and foodborne diseases associated with *S. sonnei* occur in developed countries (Hale, 1991). To date, approximately 480 strains of *S. flexneri* (NCBI genome ID 182), 113 strains of *S. boydii* (genome ID 496), 1338 strains of *S. sonnei* (genome ID 417; Fig. 4) and 67 strains of *S. dysenteriae* (genome ID 415) have been registered (Table 1). The genome of *S. flexneri* strain *2a str. 301* was the first to be obtained (Jin *et al.*, 2002). *S. flexneri* serotype 2a and *E. coli* K12 MG1655 share a high degree of genomic similarity (Stephens and Murray, 2001; Yang *et al.*, 2005). They exhibit a common sequence of approximately 3 Mb (65% in *E. coli*) that encodes 2790 proteins. In PCR‐based detection assays, specific gene regions of *ipaH*, *virA*, *ial* and *16S rRNA* have been used as targets for *Shigella* genus identification (Warren *et al.*, 2006). The *ipaH* gene, encoding the invasive plasmid antigen H, is carried by four *Shigella* species (Dutta *et al.*, 2001; Warren *et al.*, 2006). Specific regions of the *rfc* gene of *S. flexneri*, the *wbgZ* gene of *S. sonnei* and the *rfpB* gene of *S. dysenteriae* have been used to differentiate the three *Shigella* species (Ojha *et al.*, 2013). SSR markers that can distinguish *Shigella* species have also been identified (Sahl *et al.*, 2015). A recent study differentiated all four *Shigella* species by performing multiplex PCR analysis of differentiated genes (Kim *et al.*, 2017a), for which a putative restriction endonuclease gene specific to *S. sonnei*, a hypothetical protein gene specific to *S. boydii* and *S. dysenteriae* and a repressor protein gene specific to *S. flexneri* were used*.*

The *Campylobacter* genus includes two major species of foodborne pathogens, *C. jejuni* and *C. coli.* To date, about the genomes of 1615 strains of *C. jejuni* have been registered (Fig. 4), and nearly 100 representative references describing genomic research have been summarized (NCBI genome ID 149). The genome of *C. jejuni* is relatively small, with a low GC% (Table 1). The genome of the representative *C. jejuni* strain NCTC 11168 has been reported (Parkhill *et al.*, 2000), and has been indicated to harbour only a few repeat sequences and no transposons, phage remnants or insertion elements (Parkhill *et al.*, 2000; Dorrell *et al.*, 2001). *C. coli* is another pathogen that shows a distinctive epidemiology (Gillespie *et al.*, 2002). To date, 928 genomic sequences have been registered for *C. coli*, and nearly 40 representative references have been summarized (NCBI genome ID 1145). The *hip*, *16S rRNA*, *rrs*, *cdaF*, *porA*, *Hyp*, *cjaA*, *ceuE*, *hipO*, *mapA*, *ceuA*, *askD*, *glyA*, *lpxA*, *ccoN*, *ORF‐C sequence*, *rpoB*, *oxidoreductase gene*, *cdtA* and *pepT* genes are widely used for the PCR identification of *C. jejuni* (Frasao *et al.*, 2017). The other related genetic regions used for *C. coli* identification are summarized in Table 1.

### WGS and genetic marker identification of related phytopathogenic fungi

**Fig. 5:**
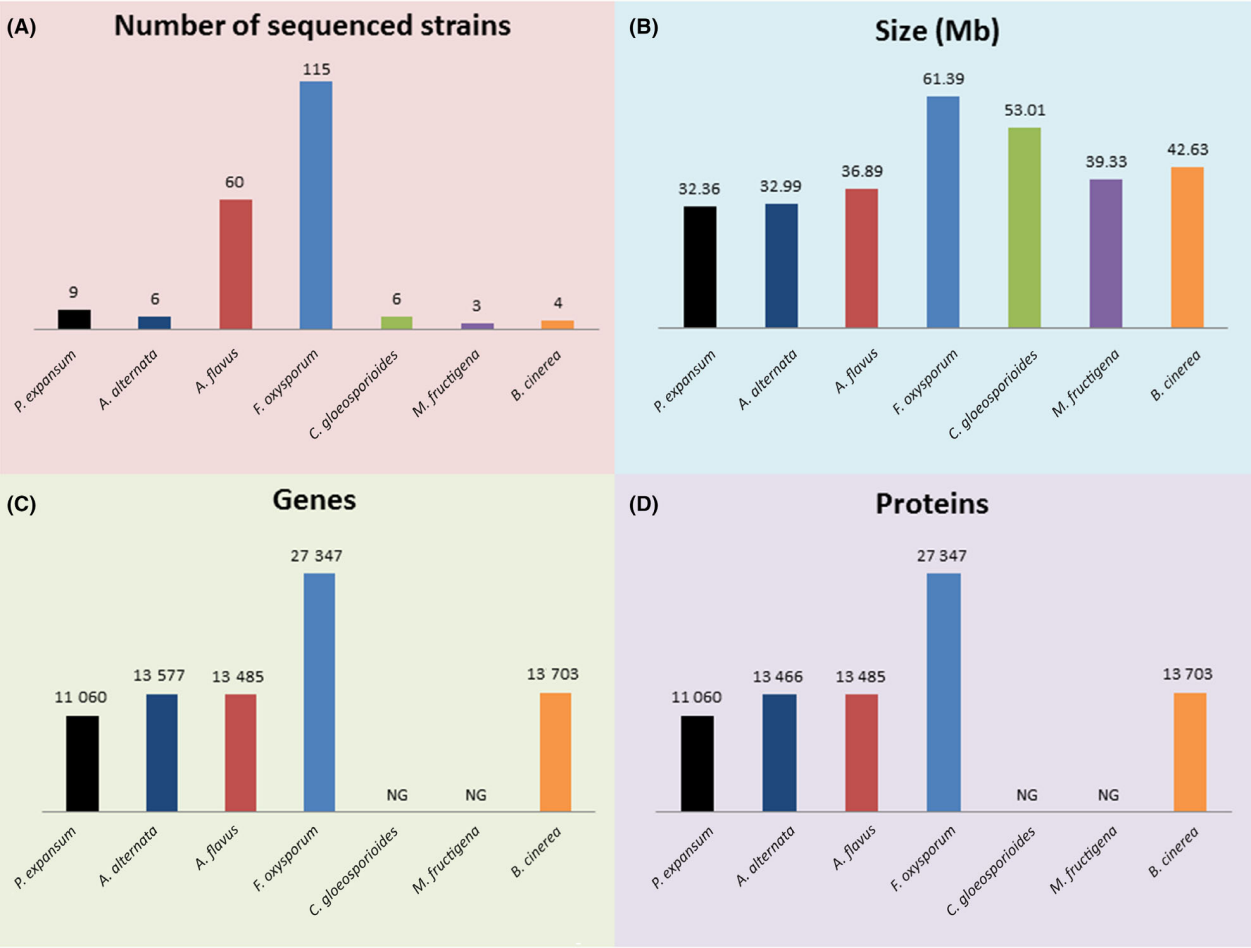
Genomic information for major phytopathogenic fungal species of *Penicillium expansum* (*P. expansum*), *Alternaria alternata* (*A. alternate*), *Aspergillus flavus* (*A. flavus*), *Fusarium oxysporum* (*F. oxysporum*), *Colletotrichum gloeosporioides* (*C. gloeosporioides*), *Monilinia fructicola* (*M. fructicola*) and *Botrytis cinerea* (*B. cinerea*).

The *Penicillium* genus contains several pathogenic species, particularly *P. expansum*, *P. digitatum*, *P. griseofulvum*, *P. italicum* and *P. citrinum.* The majority of these species are related to the postharvest decay of F & V. *P. chrysogenum* was the first sequenced species in this genus, as it is used as an industrial penicillin producer (van den Berg *et al.*, 2008). *P. expansum* is an important pathogen that accelerates corruption in various produce species (Nie, 2017; Shen *et al.*, 2018a). *P. expansum* also produces the mycotoxins patulin and citrinin. *P. expansum* strain R19 was the first to be sequenced (Yu *et al.*, 2014). To date, the genomes of nine strains of *P. expansum* have been registered (NCBI genome ID 11336; Fig. 5). A representative genome was reported for *P. expansum* from strain MD‐8 (Ballester *et al.*, 2015). *P. digitatum* causes postharvest green mould in citrus fruits (Marcet‐Houben *et al.*, 2012). The genomes of two *P. digitatum* strains, PHI26 and Pd1, have been reported (Marcet‐Houben *et al.*, 2012). *P. italicum* causes postharvest blue mould on citrus fruit (Deng *et al.*, 2018). The representative strain of *P. italicum* PHI1 was reported (Ballester *et al.*, 2015). Genomic comparison showed that *P. expansum* and *P. italicum* present differences in gene clusters related to secondary metabolism (Ballester *et al.*, 2015; Li *et al.*, 2015). Fifteen genes for patulin biosynthesis have been identified in *P. expansum*, which are located in a gene cluster (Ballester *et al.*, 2015; Li *et al.*, 2015). These genes and functions have been reviewed previously (Puel *et al.*, 2010). Several methods, including RAPD (Schena *et al.*, 2000), SNP (Piombo *et al.*, 2018) and microsatellite analysis (Mohmed *et al.*, 2010), have been used for distinguishing *Penicillium* species. The isoepoxydon dehydrogenase (*IDH*) gene is considered to be a useful marker for distinguishing patulin‐producing and nonproducing *Penicillium* species (De *et al.*, 2016; Rharmitt *et al.*, 2016). Several gene regions, such as the *patF* (Tannous *et al.*, 2015), *ITS* (Hammami *et al.*, 2017), *Pepg1* (Ostry *et al.*, 2018) and polygalacturonase genes (Hesham *et al.*, 2011), have been used for the identification of *P. expansum*. The specific genes employed for the identification of *P. digitatum*, *P. griseofulvum*, *P. italicum* and *P. citrinum* are summarized in Table 2.

The *Alternaria* genus contains several pathogenic species, particularly *A. alternata*, *A. arborescens*, *A. brassicicola* and *A. solani*. These pathogens commonly cause plant diseases and postharvest rot (Harteveld *et al.*, 2014). Additionally, *Alternaria* species can produce host‐specific phytotoxins (HSTs), which differ between plant species (Akamatsu *et al.*, 1999). *Alternaria* toxins are a group of mycotoxins produced by *Alternaria* species that mainly include tenuazonic acid (TeA), alternariol (AOH), alternariol monomethyl ether (AME), tentoxin (Ten) and altenuene (ALT). However, the genes responsible for *Alternaria* toxin biosynthesis have not yet been confirmed. To date, six strains of *A. alternata* have been registered (NCBI genome ID 11201; Fig. 5). Nguyen *et al.* (2016) reported the first draft genome of *A. alternata* ATCC 34957, obtained using PacBio SMRT technology, and discussed the gene regions related to mycotoxin metabolism. *A. arborescens* is another pathogen in this genus. Hu, *et al.* (2012) generated the first sequence for *A. arborescens* and demonstrated the horizontal transfer of the conditionally dispensable chromosome (CDC) carrying HST genes. The genome characteristics of *A. arborescens*, *A. brassicicola*, *A. solani* and *A. tenuissima* are summarized in Table 2. Several methods have been used for the identification of *Alternaria* species, such as high‐resolution melting (HRM) analyses, AFLP and SSR (Lorenzini and Zapparoli, 2014; Wolters *et al.*, 2018). Genes such as *histone‐3,* glyceraldehyde 3‐phosphate dehydrogenase (*Gpd*), *Alt a1*, *AaSdhB*, *AaSdhC*, *AaSdhD,* ITS and *β‐tubulin* have been used for *Alternaria* species identification (Table 2). The *Alt a1* gene is a widely used marker for *Alternaria* species (Gabriel *et al.*, 2017). The polyketide synthetase (PKS) gene and nonribosomal peptide synthesis (NRPS) gene are essential for *Alternaria* toxin synthesis and regulation and can also be used to identify *Alternaria* toxin‐producing species (Chen *et al.*, 2015).

The *Aspergillus* genus is large and contains several saprophytic/pathogenic species, particularly *A. flavus*, *A. parasiticus*, *A. carbonarius*, *A. niger*, *A. tubingensis* and *A. westerdijkiae*. These species cause corruption in various agricultural products and produce the aflatoxin and ochratoxin A mycotoxins. To date, the genomes of a total of 60 *A. flavus* strains have been registered (NCBI data, genome ID 360; Fig. 5). A representative genome of *A. flavus* was reported from strain NRRL3357 (Nierman *et al.*, 2015). Genomic comparison between *A. flavus* strains NRRL3357 and AF70 showed polymorphisms in their aflatoxin toxin gene cluster (Sharma *et al.*, 2018). *A. parasiticus* is another important aflatoxin producer. Two strains of *A. parasiticus* have been sequenced, and the representative strain is SU‐1 (NCBI genome ID 12976). Genomic comparison revealed approximately 98% similarity between the six *A. flavus* species and 81% similarity between *A. flavus* and *A. parasiticus* species (Faustinelli *et al.*, 2016). Fourteen strains of *A. niger* have been registered, the majority of which have been related to citrate production or sugar metabolism (Aguilar‐Pontes *et al.*, 2018; Laothanachareon *et al.*, 2018). *A. carbonarius* is another ochratoxin A‐producing member of this genus, for which one strain has been sequenced (NCBI genome ID 947). As illustrated in previous reviews, the genes responsible for aflatoxin biosynthesis are integrated as a cluster that contains approximately 25 genes with a total length of 80 kb (Yu *et al.*, 2004; Moore *et al.*, 2010). This gene cluster contains the main regulatory genes *aflR* and *aflS* and the biosynthesis genes *aflD*, *aflM*, *aflP*, *aflQ aflD*, *aflO* and *aflQ*. Aflatoxin biosynthesis and regulatory genes have been widely used to identify toxin‐producing species (Mahmoud, 2015; Hua *et al.*, 2018). Polymorphisms of the *calmodulin* gene have been used to identify *Aspergillus* species (Palumbo and O'Keeffe, 2015. The *β‐tubulin* gene can also be used for the specific identification of several *Aspergillus* species (Nasri *et al.*, 2015; Falahati *et al.*, 2016). The other genetic markers for *Aspergillus* species are summarized in Table 2.

The *Fusarium* genus contains several pathogenic species, particularly *F. oxysporum*, *F. fujikuroi*, *F. verticillioides*, *F. proliferatum*, *F. graminearum* and *F. sporotrichioides.* These pathogens cause serious diseases in crops and vegetables and produce toxic trichothecene mycotoxins. The genomes of a total of 115 *F. oxysporum* strains have been registered (NCBI genome ID 707; Fig. 5). The representative strain of *F. oxysporum* f. sp. lycopersici 4287 has been reported (Ma *et al.*, 2010). Genome comparison between *F. graminearum*, *F. verticillioides* and *F. oxysporum* revealed genomic lineage‐specific (LS) regions in the *Fusarium* genus (Ma *et al.*, 2010). *F. fujikuroi* is a plant pathogen that causes bakanae disease in rice and produces gibberellins (GAs). A total of 15 strains of this pathogen have been sequenced (NCBI genome ID 13188). The representative *F. fujikuroi* strain IMI 58289 has been reported (Wiemann *et al.*, 2013). *F. proliferatum* is also a plant pathogen, and the genomes of 13 strains of this species have been registered (NCBI genome ID 2434). The representative strain *F. proliferatum* ET1 has been reported (Niehaus *et al.*, 2016). For genetic detection, the *Fusarium*‐specific gene regions that have been used have mainly included the translation elongation factor‐1α (*tef‐1α*) gene (Wu *et al.*, 2016), *ITS* (Jedidi *et al.*, 2018), *SIX* (Debbi *et al.*, 2018) and *FUM* gene (Omori *et al.*, 2018) sequences. The genetic markers used for *F. graminearum* and *F. sporotrichioides* are summarized in Table 2.

The *Colletotrichum* genus contains several pathogenic species, particularly *C. gloeosporioides*, *C. acutatum*, *C. coccodes* and *C. fructicola*. *C. gloeosporioides* infects many crops, causing anthracnose diseases. A total of six strains of *C. gloeosporioides* have been registered, among which the representative strain is *C. gloeosporioides* SMCG1#C (NCBI genome ID 17739; Fig. 5). *C. acutatum* widely infects F & V (Cano *et al.*, 2004; Heilmann *et al.*, 2006). Two strains of this species have been registered (NCBI genome ID 38530). In addition, the genomes of two strains of *C. coccodes* and three strains of *C. fructicola* have been registered (Table 2). However, they were only registered at the scaffold level, and no related references have been reported. For PCR detection, the *ITS*, *β‐tubulin*, *actin*, *act*, *ApMat*, *cal*, *chs1*, *gapdh*, *gs*, *his3*, *tub2*, *glyceraldehyde‐3‐phosphate dehydrogenase* and *coxidase subunit 1* gene have been widely used (Tapia‐Tussell *et al.*, 2008; Chung *et al.*, 2010; Ramdeen and Rampersad, 2013; Yang *et al.*, 2015; Sharma *et al.*, 2017).

The *Monilinia* genus contains several species related to brown rot on F & V, particularly *M. laxa*, *M. fructicola*, *M. fructigena* and *M. polystroma.* However, the genomes of *Monilinia* species have rarely been reported. To date, 2 strains of *M. laxa*, 2 strains of *M. fructicola*, 3 strains of *M. fructigena* (Fig. 5) and 1 strain of *M. polystroma* have been registered. The representative *M. laxa* strain 8L has been reported (NCBI data, genome ID 66927; Naranjo‐Ortiz *et al.*, 2018). The inter‐simple sequence repeat (ISSR), RAPD (Fazekas *et al.*, 2014) and HRM (Papavasileiou *et al.*, 2016) methods have been used for interspecies identification. Specific regions of the *cytochrome b* gene (Ortega *et al.*, 2019), *laccase‐2* gene (Wang *et al.*, 2018b), *β‐tubulin* gene (Fan *et al.*, 2014), *ITS* (Guinet *et al.*, 2016), *MO368‐1*, *Laxa* (Cote *et al.*, 2004) and small subunit (*SSU*) rDNA (18S) gene (Fulton and Brown, 1997) have been reported as markers for PCR‐based detection.

The *Botrytis* genus contains the pathogenic species *B. cinerea*. *B. cinerea* causes serious grey mould diseases on F & V (Reich *et al.*, 2016). Four strains of *B. cinerea* have been registered, among which the representative strain is B05.10 (NCBI genome ID 494; Fig. 5). Genomic comparative analysis between *B. cinerea* and *Sclerotinia sclerotiorum* revealed extensive genetic polymorphisms, but showed few significant polymorphisms in specific pathogenic clusters (Amselem *et al.*, 2011). The RAPD and HRM methods have been used to identify *B. cinerea* (Thompson and Latorre, 1999). The genetic markers used for *B. cinerea* include the *ITS* (Reich *et al.*, 2016), the necrosis and ethylene‐inducing protein gene (Munoz *et al.*, 2016), the *Bc‐hch* locus (Zhang *et al.*, 2018), *G3PDH*, *HSP60* and *RPB2* (Zhou *et al.*, 2014), the necrosis and ethylene‐inducing protein 1 gene (Fan *et al.*, 2015), the species‐specific sequence‐characterized amplified region (*SCAR*) marker (Suarez *et al.*, 2005) and the intergenic spacer (*IGS*) region (Diguta *et al.*, 2010).

## 16S rDNA and ITS sequencing of the microbiome community on F & V

### 16S rDNA and ITS sequencing‐based metagenomics

**Fig. 6:**
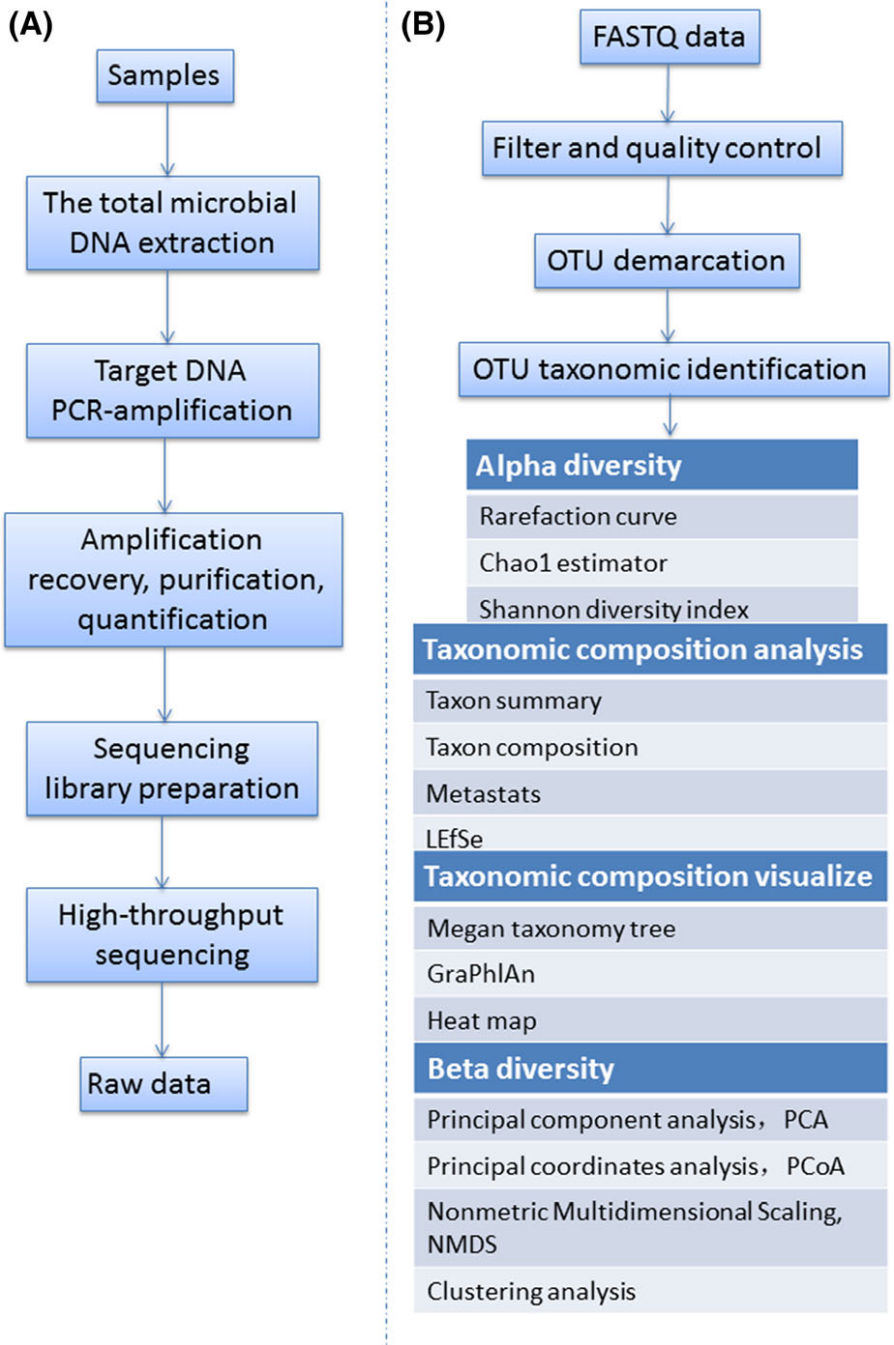
Basis of metagenomic analyses. General workflow for NGS‐based metagenomics (A). Bioinformatic analysis workflow for metagenomic data analysis (B).

Metagenomic strategies are technological approaches that are increasingly being used to study the overall microbial community in complex biological samples (Cao *et al.*, 2017). Significantly, metagenomics expands the scope of microbiology research and provides new insights into uncultivable microbes. This method is mainly based on polymorphisms in the bacterial 16S rDNA and fungal ITS regions, combined with powerful sequencing technologies, databases and software platforms (Fig. 6A). Total microbial DNA is directly extracted from research samples under this approach. rDNA PCR‐based denaturing gradient gel electrophoresis (PCR‐DGGE) was the first method developed to identify differential abundant microbes at a general level (Wang *et al.*, 2015). The distinguished rDNA fragments are obtained by gel cutting, followed by sequencing. Then, the microbes are identified by performing sequence alignment against databases. In the 2000s, NGS could be used to sequence all of the obtained rDNA amplifications, which allows the microbial community to be analysed more deeply and comprehensively. The obtained sequences are assigned to operational taxonomic units (OTUs) based on similarity (Caporaso *et al.*, 2010). Representative OTU sequences are identified (Fig. 6B). Additionally, OTU abundance provides relatively quantitative information for specific microbial taxa. Several analytical software platforms, such as the FLASH and QIIME packages, are used for downstream data analysis (Jünemann *et al.*, 2017). To address the short read length and PCR dependence of NGS, TGS‐based metagenomics is promising and powerful (Uyaguari‐Diaz *et al.*, 2016). The long reads obtained via TGS can be used to directly identify microbes at the species or even strain level. Additionally, the sequence abundance accurately represents the number of specific microbes. Once the cost is reduced, TGS will become a powerful tool for metagenomic research.

Many studies have elucidated the bacterial and fungal communities on F & V by using 16S rDNA and ITS sequencing. For this review, we searched references mainly from the Web of Science, NCBI, ScienceDirect and CNKI platforms. A total of 64 original studies describing the microbiomes of various F & V were identified (Table 3). Illumina sequencing and Roche 454 pyrosequencing have been most widely used for these studies. PCR‐DGGE combined with AB3730xl sequencing was mainly used in earlier studies. Until recently, no TGS‐based metagenomic analyses of F & V had been reported. Based on common themes, we grouped these references into three categories: microbiome diversity between plant species/genotypes; regional/environmental factors and farming practices affecting microbiomes; and microbiomes affected by artificial treatment and quality control procedures in storage and processing.

### Microbiome diversity between plant species/genotypes

**Fig. 7:**
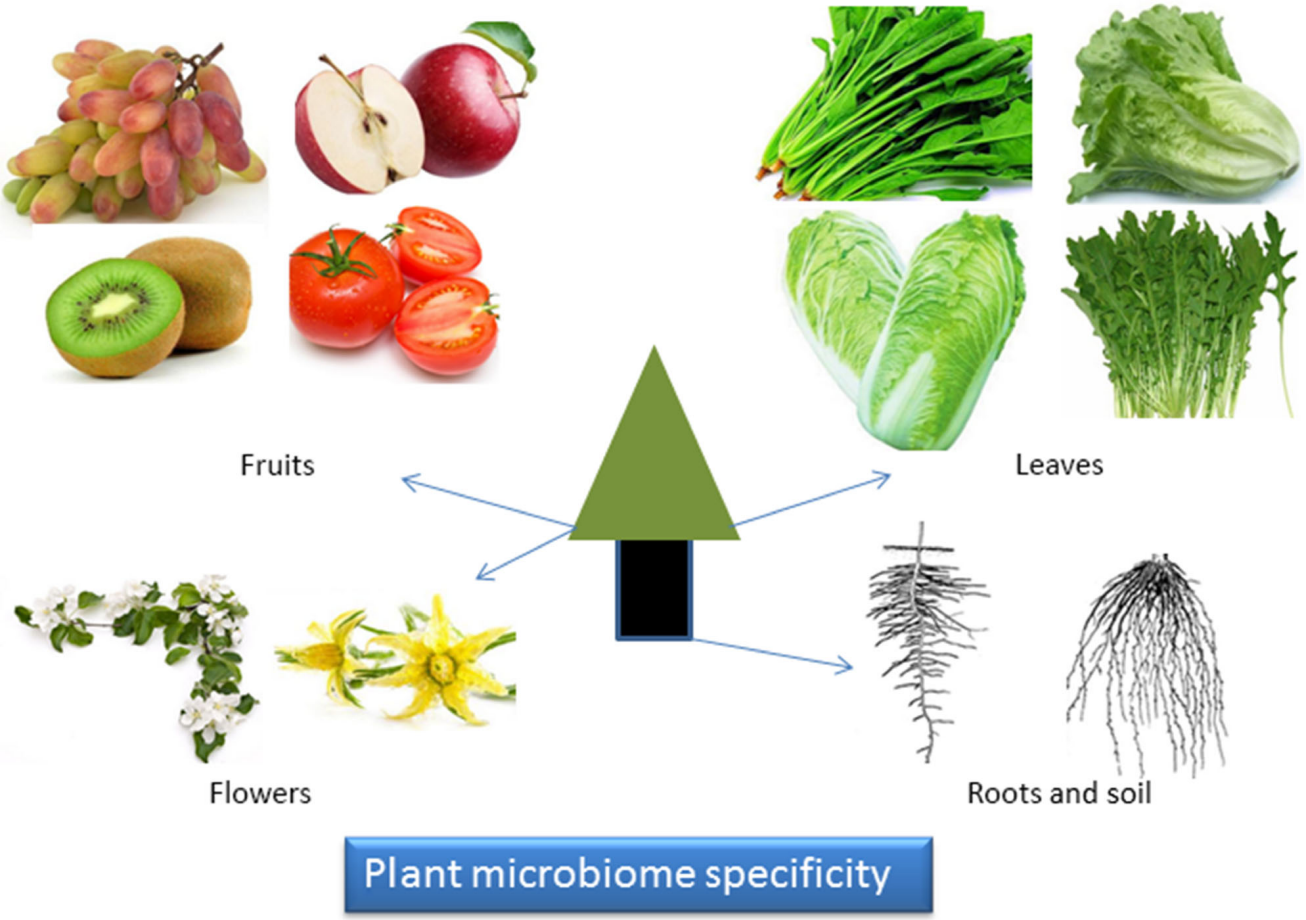
Microbiome specificity in various plant species and tissues.

Plant microbiomes are related to species/genotype specificity (Fig. 7). Recently, differential bacterial and fungal communities have been recorded on diverse fruits, including apples (Soliman *et al.*, 2015), blueberries (Jiang *et al.*, 2017), grapes (Pinto *et al.*, 2014; Zhang *et al.*, 2017a), kiwifruits (Purahong *et al.*, 2018), spinach (Darlison *et al.*, 2019) and tomatoes (Ottesen *et al.*, 2013). Because of the importance of leafy vegetables in food control, the phyllosphere microbiomes have been monitored on vegetables such as spinach (Chen *et al.*, 2018), lettuce (Higgins *et al.*, 2018) and rocket (Darlison *et al.*, 2019). In addition, the microbial community may be related to the specificity of plant tissue. The microbiomes differ among plant organs such as the fruits, leaves, flowers (Shade *et al.*, 2013) and roots (Ottesen *et al.*, 2013; Zhang *et al.*, 2017b). However, they also show correlations between several plant tissues (Zhang *et al.*, 2017b). The microbial community on F & V products is related to different characteristics and biological processes. In particular, the bacterial communities on several wine grape varieties have been found to differ, which may affect the fermentation process in winemaking (Bokulich *et al.*, 2014). Additionally, the microbial communities of plants might be related to their chemical compositions. Recently, relationships between phyllosphere minerals and microbial communities have been observed in spinach and rocket (Darlison *et al.*, 2019). Bacterial communities present in the plant rhizosphere could potentially be used as indicators of the soil environment and mineral efficiency (Jiang *et al.*, 2017). Microbiome–host or microbiome–mineral interactions might be widespread. These NGS‐based metagenomic studies have shown that the microbiomes of F & V are plant specific.

### Microbiomes affected by regional/environmental factors and farming practices

**Fig. 8:**
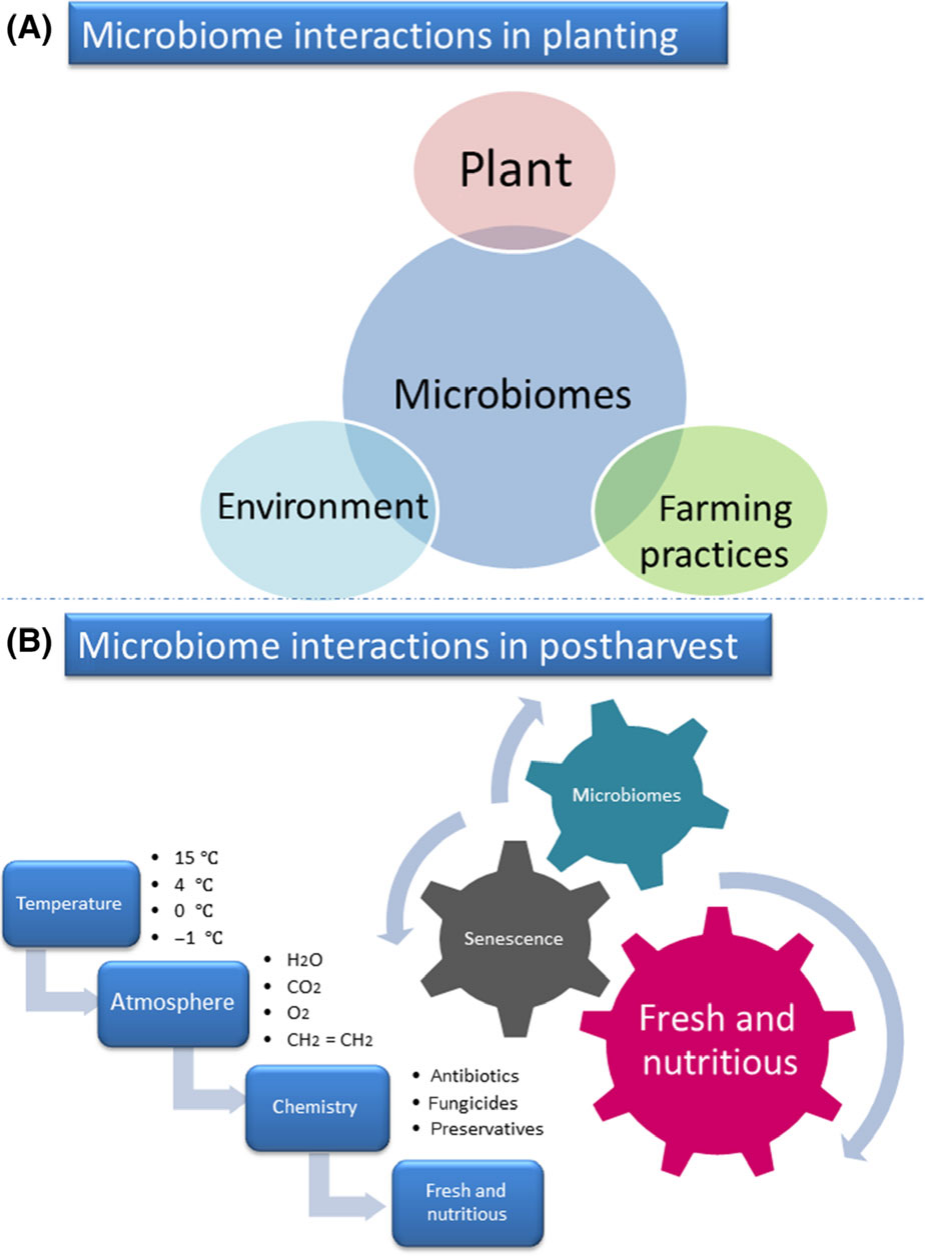
Microbiomes related to regional/environmental factors and farming practices (A). Microbiomes related to postharvest processing and storage (B).

The microbiomes of F & V are affected by differences in regional environments, farming practices and disease occurrences (Fig. 8A). Many environmental factors influence microbial activities, including temperature, humidity, light exposure, soil organic matter and mineral compositions (Yu *et al.*, 2016; Higgins *et al.*, 2018). Regional verification based on microbial communities has been conducted on shea fruits (El Sheikha *et al.*, 2011), peaches (Bigot *et al.*, 2015) and grapes (Mezzasalma *et al.*, 2017; Mezzasalma *et al.*, 2018). Recently, a comprehensive review summarized the complex relationships between wine quality, grape microbial communities and regional climates (Droby and Wisniewski, 2018). The natural microbiomes of grapes are important for fruit maturation and wine fermentation, especially regarding the formation of some secondary metabolites related to wine colour and flavour (Mezzasalma *et al.*, 2017). Agricultural inputs and farming practices such as fertilizers and pesticides influence the formation of microbial communities on agricultural products (Allard *et al.*, 2018; Chen *et al.*, 2018). Fertilization has been reported to be a factor affecting the plant microbiomes of maize plants (Chen *et al.*, 2018). The products and soil associated with organic and conventional farming practices show differences in their microbiome communities. Specifically, F & V such as grapes (Mezzasalma *et al.*, 2017), apples (Abdelfattah *et al.*, 2016), peaches (Bigot *et al.*, 2015), lettuce and spinach (Leff and Fierer, 2013) produced from organic and conventional agriculture systems have been observed to exhibit differences in their microbial communities. However, the significant relationships between microbiomes and the regional environment and farming practices are only just beginning to be revealed.

### Effects of artificial treatments in storage and processing on microbiomes

The treatments applied to F & V during postharvest processing and storage affect microbial communities (Fig. 8B). Refrigerated storage is a common approach that is considered to keep food fresh and nutritious. During cold storage, the microbial composition and activity are altered. Variations in microbial communities during cold storage have been reported in apples (Shen *et al.*, 2018a), lettuce and spinach (Lopez‐Velasco *et al.*, 2011). Different fungal structures have been identified between harvest‐point and stored samples (Shen *et al.*, 2018a). Compared with the harvest‐point samples, stored apples show increases in the relative abundance of several genera, particularly *Aspergillus*, *Botrytis*, *Mucor* and *Penicillium*. The spinach phyllosphere was observed to present a decrease in bacterial diversity after 15 days of storage at 4°C or 10°C, which might be related to the inhibition of bacterial activity by the lower temperature (Lopez‐Velasco *et al.*, 2011). In addition, physical and chemical treatments affect the microbial communities of postharvest F & V. Physical gamma irradiation treatment of romaine lettuce alters the leaf bacterial community and reduces the survival rate and regrowth ability of pathogenic bacteria (Dharmarha *et al.*, 2019a). High‐hydrostatic pressure treatments change the dynamics of the overall bacterial population and extend the shelf life of sweet cherries and asparagus (del Arbol *et al.*, 2016a, b). Antibiotics and fungicides have been shown to alter the microbial communities and extend the shelf life of several F & V. Hypochlorite treatment alters the structure of bacterial communities and extends the shelf life of carrots (Dharmarha *et al.*, 2019b). The application of fungicides alters the dynamics of yeast communities on grape berries (Milanovic *et al.*, 2013). To improve postharvest storage, the relationships between the postharvest treatment of F & V and their microbial communities require more attention.

## Conclusion

The development of DNA sequencing technology has provided an effective method for microbial WGS and genetic analysis. In recent decades, DNA sequencing technologies have been successfully developed including approximately ten operational platforms. By performing DNA sequencing, microbial WGS is largely promoted. Genetic studies provide DNA markers for microbial identification at the genetic level. These markers are extensively used in PCR or chip‐based detection. On the basis of 16S rDNA and ITS sequencing, metagenomic approaches are now emerging technologies for analysing the entire microbial community in a complex F & V matrix. The microbiomes of F & V show huge differences between plant species/genotypes. In addition, the microbiomes of F & V are related to factors such as regional/environmental factors, farming practices and postharvest treatments. These studies shed light on ways to improve F & V cultivation, disease prevention and quality control.

## Conflict of interest

The authors declare no conflict of interest.
